# *SEC16A* Variants Predispose to Chronic Pancreatitis by Impairing ER‐to‐Golgi Transport and Inducing ER Stress

**DOI:** 10.1002/advs.202402550

**Published:** 2024-08-09

**Authors:** Min‐Jun Wang, Yuan‐Chen Wang, Emmanuelle Masson, Ya‐Hui Wang, Dong Yu, Yang‐Yang Qian, Xin‐Ying Tang, Shun‐Jiang Deng, Liang‐Hao Hu, Lei Wang, Li‐Juan Wang, Vinciane Rebours, David N. Cooper, Claude Férec, Zhao‐Shen Li, Jian‐Min Chen, Wen‐Bin Zou, Zhuan Liao

**Affiliations:** ^1^ Department of Gastroenterology Shanghai Institute of Pancreatic Diseases Shanghai Key Laboratory of Nautical Medicine and Translation of Drugs and Medical Devices Changhai Hospital National Key Laboratory of Immunity and Inflammation Naval Medical University Shanghai 200433 China; ^2^ Department of Cell Biology Center for Stem Cell and Medicine Naval Medical University Shanghai 200433 China; ^3^ Inserm EFS UMR 1078 GGB Univ Brest Brest F‐29200 France; ^4^ Service de Génétique Médicale et de Biologie de la Reproduction CHRU Brest Brest F‐29200 France; ^5^ Center for Translational Medicine Naval Medical University Shanghai 200433 China; ^6^ Department of Prevention and Health Care Eastern Hepatobiliary Surgery Hospital Naval Medical University Shanghai 200438 China; ^7^ Pancreatology and Digestive Oncology Department Beaujon Hospital APHP – Clichy Université Paris Cité Paris 92110 France; ^8^ Institute of Medical Genetics School of Medicine Cardiff University Cardiff CF14 4XN United Kingdom

**Keywords:** disease predisposition, endoplasmic reticulum stress, pancreatitis, protein vesicle trafficking, SEC16A

## Abstract

Chronic pancreatitis (CP) is a complex disease with genetic and environmental factors at play. Through trio exome sequencing, a *de novo SEC16A* frameshift variant in a Chinese teenage CP patient is identified. Subsequent targeted next‐generation sequencing of the *SEC16A* gene in 1,061 Chinese CP patients and 1,196 controls reveals a higher allele frequency of rare nonsynonymous *SEC16A* variants in patients (4.90% vs 2.93%; odds ratio [OR], 1.71; 95% confidence interval [CI], 1.26–2.33). Similar enrichments are noted in a French cohort (OR, 2.74; 95% CI, 1.67–4.50) and in a biobank meta‐analysis (OR, 1.16; 95% CI, 1.04–1.31). Notably, Chinese CP patients with *SEC16A* variants exhibit a median onset age 5 years earlier than those without (40.0 vs 45.0; *p* = 0.012). Functional studies using three CRISPR/Cas9‐edited HEK293T cell lines show that loss‐of‐function *SEC16A* variants disrupt coat protein complex II (COPII) formation, impede secretory protein vesicles trafficking, and induce endoplasmic reticulum (ER) stress due to protein overload. *Sec16a* ^+/−^ mice, which demonstrate impaired zymogen secretion and exacerbated ER stress compared to *Sec16a* ^+/+^, are further generated. In cerulein‐stimulated pancreatitis models, *Sec16a* ^+/−^ mice display heightened pancreatic inflammation and fibrosis compared to wild‐type mice. These findings implicate a novel pathogenic mechanism predisposing to CP.

## Graphical abstract

SEC16A is a crucial regulator in the formation of COPII vesicles, mediating protein transport from ER to Golgi apparatus. Here, a link between rare *SEC16A* variants and chronic pancreatitis, demonstrating that loss of SEC16A function disrupts ER‐to‐Golgi trafficking of secretory proteins in pancreatic acini is identified. This disruption leads to protein overload‐induced ER stress, thereby predisposing individuals to the disease.

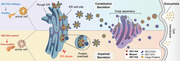

## 1 Introduction

Chronic pancreatitis (CP) is a progressive fibroinflammatory disease of the pancreatic tissue, resulting from the intricate interplay of genetic and environmental factors.^[^ ^1^ ^]^ Both the incidence and prevalence of CP have been increasing in most developed countries, with an estimated global incidence of about 10 cases per 100 000 person‐years.^[^ ^2^ ^]^ Since the pivotal discovery in 1996,^[^ ^3^ ^]^ genetic studies over the past 27 years have yielded unique insights into the molecular mechanisms underlying CP, exemplified by the identification of distinct pathogenic pathways involving trypsin‐dependent processes and the induction of endoplasmic reticulum (ER) stress.^[^ ^4^, ^5^ ^]^

Irrespective of the specific pathogenic pathways involved, most of the CP susceptibility genes reported to date encode pancreatic digestive proteases or their inhibitors. These include cationic trypsinogen (protease, serine, 1; *PRSS1*),^[^ ^3^ ^]^ serine protease inhibitor Kazal type 1 (*SPINK1*),^[^ ^6^ ^]^ chymotrypsinogen C (*CTRC*),^[^ ^7^ ^]^ carboxypeptidase A1 (*CPA1*),^[^ ^8^ ^]^ carboxyl ester lipase (*CEL*),^[^ ^9^ ^]^ chymotrypsinogen B1 and B2 (*CTRB1‐CTRB2*),^[^ ^10^ ^]^ and pancreatic lipase (*PNLIP*).^[^ ^11^ ^]^ However, it is important to note that some of these findings have not been consistently replicated in subsequent studies owing to ethnic differences in disease susceptibility.^[^ ^11^, ^12^, ^13^, ^14^ ^]^

More recently, functionally defective variants in the *TRPV6* gene (transient receptor potential vanilloid subfamily member 6) have been associated with CP.^[^ ^15^, ^16^ ^]^ Unlike the aforementioned CP susceptibility genes, *TRPV6* is expressed in multiple epithelial tissues, including the epididymis, placenta, prostate, and exocrine pancreas. This characteristic of expression across multiple tissues is also observed with *CFTR* (cystic fibrosis transmembrane conductance regulator), another gene associated with CP.^[^ ^17^, ^18^ ^]^ In this study, we describe the association of rare variants in another widely expressed gene, *SEC16A* (Sec16 homolog A, endoplasmic reticulum export factor), with CP across diverse populations. We then explore the underlying pathogenic mechanisms employing both cellular and mouse models.

## 2 Results

### 2.1 A *De Novo SEC16A* Frameshift Variant Identified in a Chinese Teenager with CP

Among 16 Chinese teenage CP patients subjected to trio exome sequencing, a 12‐year‐old boy with a history of recurrent acute pancreatitis was found to harbor a heterozygous *de novo* frameshift variant, c.6491_6492delTG (p.Val2164AlafsTer20), in the *SEC16A* gene. *SEC16A*, located on chromosome 9q34.3 and comprising 32 exons, encodes a protein of 2357 amino acids (mRNA reference sequence: NM_01 4866.2). The c.6491_6492delTG variant, not present in the Genome Aggregation Database (gnomAD) (https://gnomad.broadinstitute.org/) and previously unreported, likely results in a significant truncation of the SEC16A protein, eliminating amino acids 2106–2357, a critical region for its interaction with SEC23A (SEC23 Homolog A, COPII Coat Complex Component).^[^ ^19^ ^]^

Scrutiny of the gnomAD database (last accessed on August 2, 2023) revealed that predicted loss‐of‐function (pLoF) variants within the *SEC16A* gene, including nonsense, frameshift or GT‐AG splice site variants as defined by Lek et al.,^[^ ^20^ ^]^ have been subjected to moderate selection, as indicated by a LOEUF (loss‐of‐function observed/expected upper bound fraction) score of 0.32. SEC16A participates in the formation of coat protein complex II (COPII) vesicles, which play an important role in the membrane transport of folded proteins from the ER to the Golgi apparatus.^[^ ^21^ ^]^

Taking these strands of evidence together, *SEC16A* was considered to be a plausible candidate gene for CP susceptibility.

### 2.2 Excess of Rare *SEC16A* Variants Identified in the Chinese CP Discovery Cohort Compared to Controls

Next, we analyzed the entire coding sequence and exon/intron boundaries of the 32‐exon *SEC16A* gene in 1061 Chinese CP patients and 1196 controls using targeted next‐generation sequencing. Consistent with our prior study,^[^ ^12^ ^]^ we focused on rare variants (<1% global allele frequency in gnomAD as of August 2020), including i) micro‐deletions or micro‐insertions that affected canonical GT‐AG splice sites and/or coding sequence and ii) single nucleotide substitutions altering canonical GT‐AG splice sites or resulting in missense or nonsense mutations. The resulting 77 rare variants (74 missense, 1 splice site, 1 frameshift, and 1 in‐frame deletion; all confirmed by Sanger sequencing) are listed in **Table** **1**. These variants were heterozygous, except for one that was homozygous (c.5011A>G [p.Ile1671Val]).

**Table 1:** Distribution of rare *SEC16A* nonsynonymous variants in Han Chinese CP patients and controls.

Location	Nucleotide change	Amino acid change	Patients [*n* = 1061]		Controls [*n* = 1196]		CADD score
			+	%	+	%	
**Exon 3**	**c.43C>T**	**p.P15S**	**2**	**0.19**	**0**	**0**	**24.3**
**Exon 3**	**c.46C>T**	**p.P16S**	**1**	**0.09**	**0**	**0**	**24.9**
Exon 3	c.368C>T	p.P123L	2	0.19	0	0	0.01
Exon 3	c.528T>A	p.H176Q	2	0.19	2	0.17	0.01
**Exon 3**	**c.671C>T**	**p.P224L**	**2**	**0.19**	**0**	**0**	**20.9**
Exon 3	c.752C>G	p.P251R	7	0.66	10	0.84	12.9
Exon 3	c.1066G>A	p.G356R	1	0.09	1	0.08	7.5
Exon 3	c.1085T>C	p.L362P	0	0	1	0.08	4.6
Exon 3	c.1103C>T	p.A368V	1	0.09	0	0	17.7
**Exon 3**	**c.1118C>T**	**p.A373V**	**1**	**0.09**	**2**	**0.17**	**22.7**
Exon 3	c.1136G>A	p.G379E	1	0.09	1	0.08	14.1
**Exon 3**	**c.1157A>T**	**p.N386I**	**0**	**0**	**1**	**0.08**	**22.5**
Exon 3	c.1193C>T	p.A398V	2	0.19	1	0.08	11.4
Exon 3	c.1265G>A	p.S422N	2	0.19	0	0	1.2
Exon 3	c.1298A>G	p.N433S	1	0.09	1	0.08	0.0
Exon 3	c.1346C>T	p.P449L	1	0.09	0	0	0.003
Exon 3	c.1519G>A	p.G507R	1	0.09	0	0	11.5
Exon 3	c.1711G>A	p.V571I	0	0	1	0.08	0.1
Exon 3	c.2069C>T	p.P690L	3	0.28	0	0	7.7
Exon 3	c.2356G>C	p.G786R	1	0.09	1	0.08	17.6
Exon 3	c.2851C>G	p.L951V	1	0.09	0	0	12.2
Exon 3	c.2909C>T	p.A970V	0	0	1	0.08	9.3
Exon 3	c.3022G>A	p.V1008M	6	0.57	7	0.59	0
**Exon 3**	**c.3113A>C**	**p.D1038A**	**0**	**0**	**1**	**0.08**	**21.1**
Exon 3	c.3209C>T	p.P1070L	0	0	1	0.08	13.4
**Exon 3**	**c.3493_3513del**	**p.1165_1171del**	**1**	**0.09**	**0**	**0**	
Exon 3	c.3515A>G	p.Q1172R	1	0.09	1	0.08	15.9
**Exon 4**	**c.3592C>G**	**p.R1198G**	**0**	**0**	**1**	**0.08**	**22.8**
Exon 4	c.3646C>G	p.P1216A	1	0.09	0	0	12.2
**Exon 4**	**c.3653C>G**	**p.P1218R**	**1**	**0.09**	**2**	**0.17**	**23.7**
Exon 5	c.3782C>G	p.S1261C	0	0	1	0.08	18.6
**Intron 5**	**c.3803‐1G>A**		**1**	**0.09**	**0**	**0**	**35.0**
Exon 7	c.4004A>C	p.H1335P	1	0.09	2	0.17	2.1
Exon 7	c.4027G>A	p.V1343M	0	0	1	0.08	18.4
**Exon 7**	**c.4034G>A**	**p.R1345Q**	**3**	**0.28**	**2**	**0.17**	**26.2**
**Exon 7**	**c.4099C>T**	**p.R1367C**	**1**	**0.09**	**0**	**0**	**27.6**
**Exon 8**	**c.4147C>G**	**p.H1383D**	**1**	**0.09**	**0**	**0**	**23.6**
Exon 8	c.4247C>T	p.P1416L	3	0.28	0	0	9.8
**Exon 8**	**c.4249G>A**	**p.G1417S**	**2**	**0.19**	**0**	**0**	**21.3**
**Exon 8**	**c.4256C>T**	**p.P1419L**	**1**	**0.09**	**0**	**0**	**23.9**
Exon 8	c.4294A>G	p.M1432V	1	0.09	0	0	0.005
**Exon 10**	**c.4484G>A**	**p.R1495Q**	**11**	**1.04**	**3**	**0.25**	**29.6**
**Exon 12**	**c.4651G>A**	**p.V1551M**	**2**	**0.19**	**0**	**0**	**24.4**
**Exon 12**	**c.4786G>T**	**p.G1596C**	**0**	**0**	**1**	**0.08**	**24.4**
**Exon 13**	**c.4939C>G**	**p.L1647V**	**0**	**0**	**1**	**0.08**	**22.7**
**Exon 14**	**c.5011A>G**	**p.I1671V**	**2** **(1 homo)**	**0.19**	**2**	**0.17**	**23.2**
**Exon 14**	**c.5053C>T**	**p.R1685W**	**1**	**0.09**	**0**	**0**	**27.1**
**Exon 15**	**c.5152G>A**	**p.V1718I**	**0**	**0**	**1**	**0.08**	**23.2**
**Exon 17**	**c.5308C>T**	**p.P1770S**	**1**	**0.09**	**0**	**0**	**23.4**
**Exon 18**	**c.5441C>T**	**p.A1814V**	**1**	**0.09**	**0**	**0**	**24.8**
**Exon 19**	**c.5565A>C**	**p.L1855F**	**1**	**0.09**	**0**	**0**	**21.9**
**Exon 19**	**c.5575G>A**	**p.D1859N**	**1**	**0.09**	**1**	**0.08**	**31.0**
Exon 19	c.5624C>T	p.T1875M	1	0.09	0	0	8.0
Exon 20	c.5813C>T	p.P1938L	3	0.28	4	0.33	15.8
Exon 20	c.5828C>T	p.S1943L	1	0.09	0	0	3.0
Exon 20	c.5834C>T	p.P1945L	2	0.19	0	0	8.1
Exon 20	c.5839G>T	p.V1947L	4	0.38	0	0	14.6
**Exon 20**	**c.5842C>T**	**p.R1948W**	**1**	**0.09**	**2**	**0.17**	**24.6**
Exon 21	c.5888C>T	p.A1963V	2	0.19	0	0	17.7
Exon 21	c.6044A>G	p.H2015R	3	0.28	0	0	0.0
Exon 21	c.6055G>C	p.E2019Q	0	0	1	0.08	15.6
Exon 22	c.6115T>C	p.S2039P	1	0.09	1	0.08	0.1
Exon 23	c.6197C>T	p.P2066L	0	0	1	0.08	12.1
Exon 23	c.6214G>A	p.D2072N	0	0	1	0.08	0.1
Exon 24	c.6313G>A	p.G2105S	1	0.09	0	0	12.0
Exon 24	c.6353A>G	p.K2118R	0	0	1	0.08	13.7
Exon 26	c.6499G>A	p.A2167T	0	0	1	0.08	17.3
**Exon 27**	**c.6601G>A**	**p.G2201R**	**1**	**0.09**	**0**	**0**	**22.6**
**Exon 27**	**c.6616G>C**	**p.E2206Q**	**1**	**0.09**	**0**	**0**	**20.4**
**Exon 27**	**c.6628G>A**	**p.A2210T**	**0**	**0**	**2**	**0.17**	**21.7**
**Exon 27**	**c.6646G>A**	**p.A2216T**	**2**	**0.19**	**2**	**0.17**	**24.3**
**Exon 27**	**c.6691delA**	**p.T2231fs**	**1**	**0.09**	**0**	**0**	
Exon 28	c.6746C>T	p.A2249V	1	0.09	0	0	10.5
Exon 28	c.6763G>A	p.A2255T	0	0	1	0.08	5.8
**Exon 29**	**c.6806C>T**	**p.A2269V**	**1**	**0.09**	**1**	**0.08**	**23.4**
Exon 29	c.6832C>T	p.P2278S	1	0.09	0	0	10.1
**Exon 31**	**c.7000G>T**	**p.A2334S**	**0**	**0**	**1**	**0.08**	**23.2**
**Variant carriers in total** ^a)^			**99**	**9.33**	**70**	**5.85**	
**Variant (CADD score of ≥ 20) carriers in total**			**42**	**3.96**	**26**	**2.17**	

Variants with a CADD score of ≥20 are highlighted in bold.

^a)^ All variants were identified in the heterozygous state, except for one patient who was homozygous for c.5011A>G and four patients who were compound heterozygotes: two with **c.671C>T** and **c.4249G>A**, one with c.1066G>A and c.3022G>A, and one with **c.4484G>A** and c.5839G>T.

Crucially, all three non‐missense variants, namely the splice site (c.3803‐1G>A), frameshift (c.6691delA [p.Thr2231ProfsTer36]), and in‐frame deletion (c.3493_3513del [p.Leu1165_Pro1171del]) variants, were exclusively found in CP patients. These variants are inherently most likely to be of pathological significance. The splice site variant disrupts the canonical splice acceptor site of intron 5, likely causing a frameshift. The frameshift variant presumably leads to a non‐functional protein due to the loss of the 126 C‐terminal amino acids, crucial for SEC16A's interaction with SEC23A.^[^ ^19^ ^]^ The in‐frame deletion, removing 7 amino acids within a critical region (encompassing amino acids 1102–1405) for ER localization,^[^ ^19^ ^]^ likely impacts protein structure and function. Additionally, compound heterozygous *SEC16A* missense variants were identified in four patients but not in controls (Table 1), further suggesting a role for *SEC16A* in CP.

**Figure 1:**
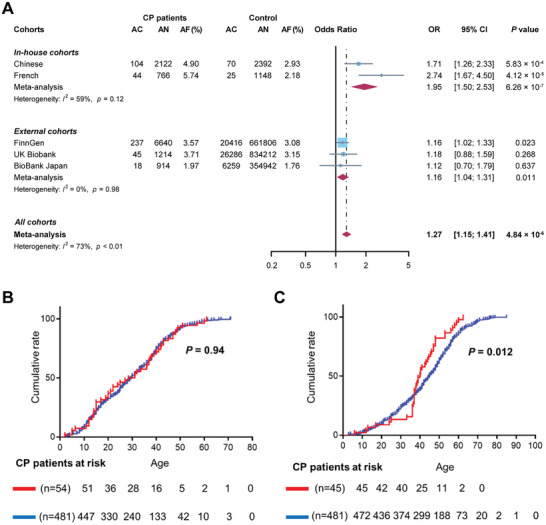
Association of rare *SEC16A* variants with CP and influence on age of onset. A) Forest plots illustrating the distribution of rare *SEC16A* variant alleles across multiple cohorts. This includes the Chinese discovery cohort, the French replication cohort, and three publicly available biobanks. Additionally, meta‐analytic data combining these various sources are presented. AC, allele count; AN, allele number; AF, allele frequency; OR, odds ratio; CI, confidence interval. B) Kaplan–Meier plots showing the impact of rare *SEC16A* variants on the age of onset of pancreatitis symptoms in Chinese patients with known pathogenic *SPINK1*, *PRSS1*, *CTRC* and/or *CFTR* genotypes. The red curve represents patients carrying rare *SEC16A* variants, whereas the blue curve denotes patients without these variants. C) Kaplan–Meier plots depicting the influence of rare *SEC16A* variants on the age of onset of pancreatitis symptoms in Chinese patients who lack known pathogenic *SPINK1*, *PRSS1*, *CTRC* and/or *CFTR* genotypes. Consistent with panel B, the red curve depicts patients with rare *SEC16A* variants whereas the blue curve represents those without these variants.

To explore the association between *SEC16A* variants and CP, we first performed an aggregate analysis using all identified rare variants in the Chinese patients versus controls (Table 1). Rare *SEC16A* variants were more prevalent in patients (99/1061, 9.33%) than in controls (70/1196, 5.85%), signifying a significant over‐representation (odds ratio [OR], 1.66; 95% confidence interval [CI], 1.20–2.26; *p* = 1.73 × 10^−3^). The allele frequency was also higher in patients (104/2122, 4.90%) as compared to controls (70/2392, 2.93%) (OR, 1.71; 95% CI, 1.26–2.33; *p* = 5.83 × 10^−4^; **Figure** **1**A).

We further classified the *SEC16A* missense variants into two subcategories based on Combined Annotation‐Dependent Depletion (CADD) predictions, using a score of 20 as the cut‐off value (generally, a CADD score of 20 is held to denote that the variant is among the top 1% of deleterious variants in the human genome). The c.6691delA frameshift variant and the c.3493_3513del in‐frame deletion variant were assigned an honorary CADD score of ≥20 on the basis of their likely severe phenotypic consequences. This classification revealed a significant over‐representation of variants with a CADD score of ≥20 among patients (42/1061, 3.96%) compared to controls (26/1196, 2.17%) (OR, 1.86; 95% CI, 1.13–3.01; *p* = 0.013); allele frequency analysis also showed a significant difference (CP versus control: 2.12% versus 1.09%; OR, 1.97; 95% CI, 1.23–3.17; *p* = 5.34 × 10^−3^) (Table 1; Figure S1, Supporting Information).

### 2.3 Rare *SEC16A* Variants Associated with CP in Replication Cohorts

We further sequenced the *SEC16A* gene in a French cohort using the above described variant inclusion criteria. Rare nonsynonymous *SEC16A* variants (all missense and heterozygous) were found at similar frequencies in idiopathic (10.94% [21/192]) and familial/hereditary (12.04% [23/191]) CP patients (Table S1, Supporting Information). Therefore, the two datasets were merged into a single dataset: rare nonsynonymous *SEC16A* variants were found in 11.49% (*n* = 44) of the 383 French patients. By contrast, rare nonsynonymous *SEC16A* variants were found only in 4.36% (*n* = 25) of the 574 French Exome (FREX) subjects (Table S2, Supporting Information). Using the FREX data as a control, rare nonsynonymous *SEC16A* variants were associated with CP in the French population (OR, 2.85; 95% CI, 1.70–4.76; *p* = 2.92 × 10^−5^). Allele frequency analysis corroborated this (CP versus control: 5.74% versus 2.18%; OR, 2.74; 95% CI, 1.67–4.50; *p* = 4.12 × 10^−5^) (Figure 1A). Variants with a CADD score of ≥20 were also more prevalent in French CP patients in terms of both carrier frequency (OR, 2.92; 95% CI, 1.29–6.78; *p* = 7.44 × 10^−3^) and allele frequency (OR, 2.87; 95% CI, 1.29–6.62; *p* = 7.86 × 10^−3^) (Figure S1, Supporting Information). Pooled ORs for rare nonsynonymous *SEC16A* variant alleles in our *in‐house* Chinese and French cohorts were 1.95 (95% CI, 1.50–2.53; *p* = 6.26 × 10^−7^) (Figure 1A) and 2.18 (95% CI, 1.43–3.30; *p* = 2.58 × 10^−4^) for those with a CADD score of ≥20 (Figure S1, Supporting Information), respectively.

Additionally, we analyzed data from three external CP cohorts in publicly available biobanks (FinnGen, UK Biobank, and BioBank Japan) for further replication. The allele frequencies of rare nonsynonymous *SEC16A* variants in CP patients were 3.57% (237/6640) in FinnGen, 3.71% (45/1214) in UK Biobank, and 1.97% (18/914) in BioBank Japan (Table S3, Supporting Information). A significant over‐representation in patients compared to controls was found in FinnGen (OR, 1.16; 95% CI, 1.02–1.33; *p* = 0.023) and in a meta‐analysis of all three external cohorts (OR, 1.16; 95% CI, 1.04–1.31; *p* = 0.011) (Figure 1A).

The combined OR for *SEC16A* variant alleles in both *in‐house* and external cohorts was 1.27 (95% CI, 1.15–1.41; *p* = 4.84 × 10^−6^) (Figure 1A), with a similar OR for variants with a CADD score of ≥20 (Figure S1, Supporting Information).

### 2.4 Rare *SEC16A* Variants Significantly Influenced Age of Onset in Genetically Unexplained CP Cases

We previously identified rare pathogenic genotypes involving four primary CP susceptibility genes, *SPINK1*, *PRSS1*, *CTRC*, and *CFTR*, in 535 (50.42%) of the 1061 Chinese patients.^[^ ^22^ ^]^ We therefore ascertained whether the rare *SEC16A* variants were more frequently found in patients with a known pathogenic genotype or vice versa in the Chinese dataset. Fifty‐four (54.5%) of the 99 patients with rare *SEC16A* variants and 481 (50.0%) of the 962 patients without rare *SEC16A* variants were found to harbor a known pathogenic genotype, a difference that was not statistically significant (*p* = 0.39).

Next, we explored whether the rare *SEC16A* variants might influence the age of disease onset, one of the most commonly used parameters for studying genotype‐phenotype relationships. Using Kaplan–Meier analysis, we found that neither the presence nor the absence of *SEC16A* variants influenced the age of disease onset in patients who carried a known pathogenic genotype (29.7; 95% CI, 20.0–37.0 versus 29.8; 95% CI, 27.6–32.0) (Figure 1B). However, in patients who did not carry a known pathogenic genotype, the median age at onset of CP in the *SEC16A* variant‐positive patients was significantly earlier than that in the *SEC16A* variant‐negative patients (40.0; 95% CI, 37.0–44.6 versus 45.0; 95% CI, 42.5–47.0; *p* = 0.012) (Figure 1C).

### 2.5 Impaired Expression of *SEC16A* Protein and Secretion of Exogenously Expressed Pancreatic Zymogens in p.Arg1495Gln and p.Val1947Leu Cell Lines

To investigate the potential pathogenic mechanism(s) underlying the rare *SEC16A* variants, we selected three missense variants, c.4484G>A (p.Arg1495Gln), c.5839G>T (p.Val1947Leu) and c.3115C>T (p.Arg1039Cys), for functional characterization in CRISPR/Cas9‐edited HEK293 cell lines (Figure S2, Supporting Information). The variants p.Arg1495Gln and p.Val1947Leu were chosen due to their greatest degree of enrichment in Chinese cases compared to controls (Table 1). By contrast, p.Arg1039Cys (not listed in Table 1), which has a global allele frequency of 20.9% in gnomAD, showed similar allele frequencies in both 1061 Chinese patients (60/2122, 2.8%) and 1196 controls (76/2322, 3.2%).

**Figure 2:**
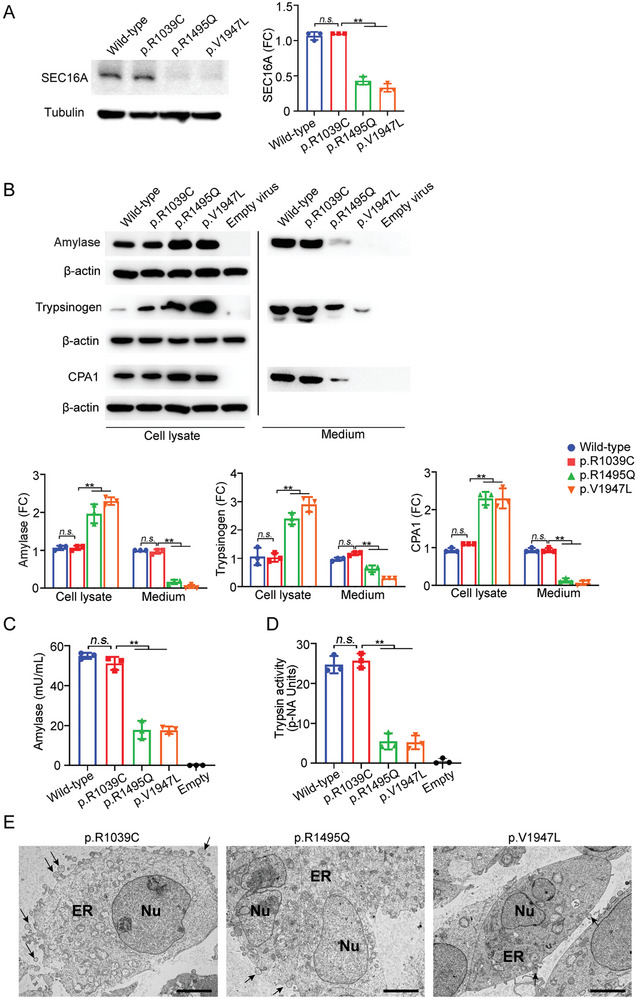
Impact of rare *SEC16A* variants on protein expression. A) SEC16A expression in CRISPR/Cas9‐edited mutant HEK293T cell lines. Left panel: A representative Western blot displaying SEC16A expression in cell lysates from both wild‐type and three HEK293T mutant cell lines. Right panel: Quantitative analysis of SEC16A expression levels in the mutant cell lines, normalized against tubulin and compared to that (set at 1) in the wild‐type cells. FC, fold change. B) Effect of rare SEC16A variants on secretion of exogenously expressed pancreatic zymogens. Upper panels: Immunoblots of cell lysates and media from both wild‐type and mutant *SEC16A* HEK293T cells expressing AMY2A, PRSS1, and CPA1 through lentiviral infection. The “Empty virus” group serves as the control, comprising uninfected HEK293T cells. Lower panels: Quantitative analysis of amylase, trypsinogen, and CPA1 expression (normalized to actin) in lysate and media of infected mutant cells versus wild‐type. Expression in wild‐type cells is set at 1. FC, fold change. C) Comparison of exogenously expressed amylase activity levels in the supernatant of mutant cell lines relative to wild‐type cells. D) Analysis of exogenously expressed trypsin activity levels in the supernatant of mutant cell lines compared to wild‐type cells. E) Ultrastructure examination of variant *SEC16A* HEK293T cells under transmission electron microscopy (TEM). Secretory vesicles are indicated with arrows. Scale bar represents 5 µm. All experiments were conducted using cell lines homozygous for the respective variants. For panels showing quantitative data, values are expressed as mean ± SD. Statistical significance is denoted as n.s. (not significant) and ^**^ (*p* < 0.01), determined using one‐way ANOVA with Tukey's multiple comparisons test.

To facilitate observation and characterization of the potential effects of these variants on SEC16A protein expression, homozygous cell lines were used. Western blot analysis of cell lysates from wild‐type and mutant HEK293T cells demonstrated that SEC16A expression was significantly reduced in the p.Arg1495Gln and p.Val1947Leu cell lines but not in the p.Arg1039Cys cell line (**Figure** **2**A). This suggested that both p.Arg1495Gln and p.Val1947Leu had a significant impact on SEC16A expression or stability.

Given the important role of SEC16A in secretory cargo trafficking from the ER to the Golgi apparatus,^[^ ^23^ ^]^ we next analyzed the potential effect of p.Arg1495Gln and p.Val1947Leu on the secretion of exogenously expressed pancreatic zymogens, using p.Arg1039Cys as a negative control. The three mutant and wild‐type HEK293T cells were each infected with *AMY2A* (Amylase Alpha 2A)‐, *PRSS1‐*, or *CPA1‐*lentivirus, followed by Western blot analysis of the exogenously expressed zymogens in both cell lysates and media. All exogenously expressed zymogens were significantly increased in lysates but significantly decreased in the media of the infected p.Arg1495Gln and p.Val1947Leu cells compared to the wild‐type. By contrast, no significant differences were observed between the p.Arg1039Cys cells and wild‐type (Figure 2B). This finding was corroborated by significantly lower amylase and trypsin activities in the media of the infected p.Arg1495Gln and p.Val1947Leu cells compared to both wild‐type and p.Arg1039Cys cells (Figure 2C,D). Transmission electron microscopy (TEM) analysis also confirmed fewer secretory vesicles in the p.Arg1495Gln and p.Val1947Leu cells compared to p.Arg1039Cys (Figure 2E). These findings indicated that the secretory function (but not the exogenous gene expression) of the HEK293 cells was impaired by the p.Arg1495Gln and p.Val1947Leu variants.

### 2.6 Impaired COPII Vesicle Formation and Increased ER Stress in p.Arg1495Gln and p.Val1947Leu Cell Lines

**Figure 3:**
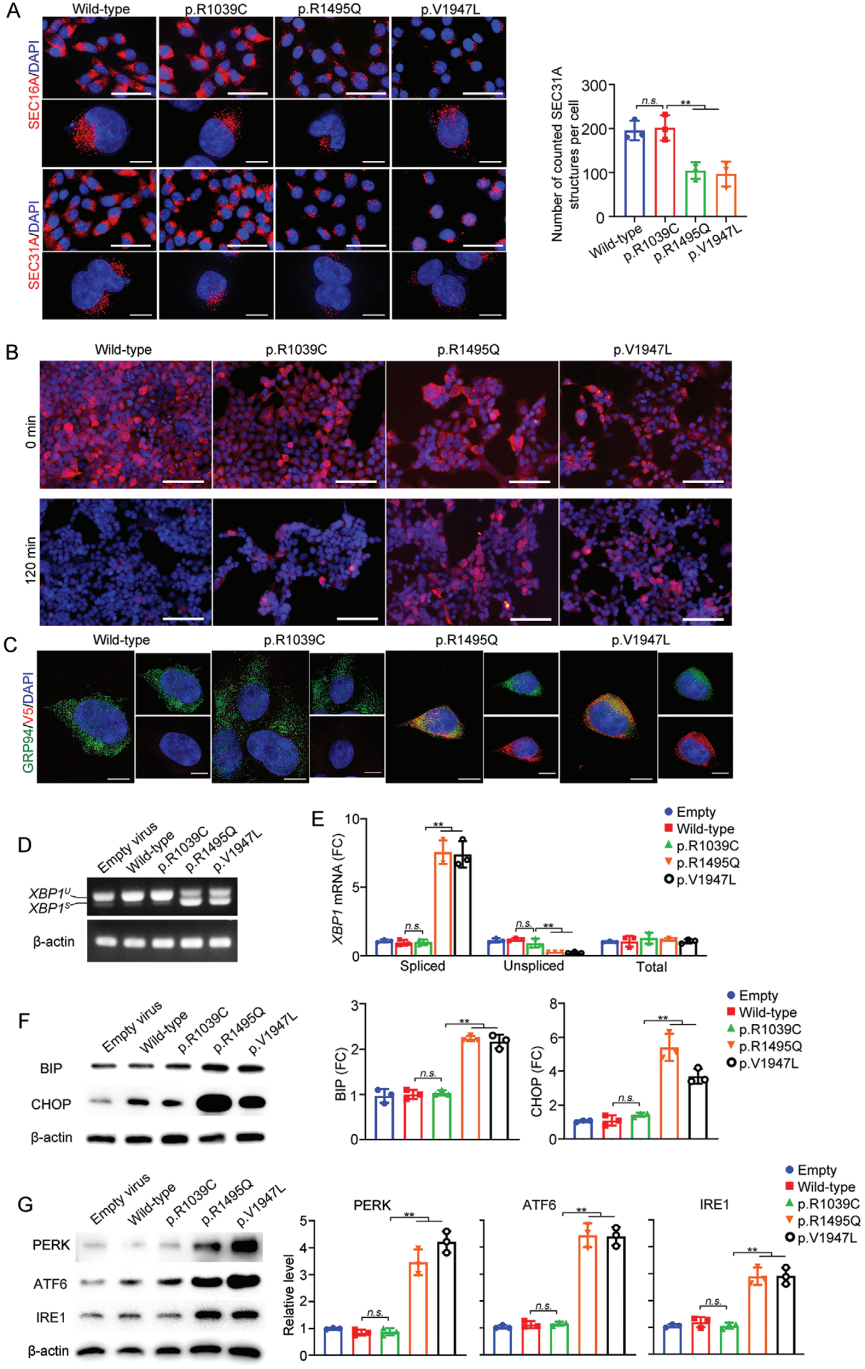
Disruption of COPII vesicle organization and ER Stress induced by *SEC16A* variants. A) Visualization of *SEC16A*‐ or SEC31A‐positive structures in CRISPR/Cas9‐edited mutant HEK293T cell lines. Left panel: Representative images of HEK293T wild‐type and *SEC16A* variant cells infected with *AMY2A* virus, immunostained for *SEC16A* or SEC31A. Scale bar: 50 µm for low‐power field and 10 µm for magnification (THUNDER Imagers, Mica). Right panel: Quantitative analysis of the average number of SEC31A‐positive structures in wild‐type versus mutant cells, based on counts from 50 cells per experiment. B) CPA1 expression in cycloheximide‐treated cells. Immunostaining of wild‐type and *SEC16A* variant HEK293T cells expressing CPA1, before and after 120 min cycloheximide treatment. Blue, DAPI staining for nuclei; red, anti‐V5 staining for CPA1. Scale bar: 100 µm. C) Higher magnification images of CPA1 expression after 120 min cycloheximide treatment (THUNDER Imagers, Mica). Blue, DAPI staining for nuclei; green, anti‐GRP94 for ER; red, anti‐V5 staining for CPA1. Scale bar: 10 µm. D) *XBP1* mRNA splicing analysis. Reverse transcription‐PCR analysis of unspliced (U) and spliced (S) *XBP1* mRNA in wild‐type and *SEC16A* mutant cells. E) Quantitative real‐time PCR of *XBP1* mRNA. Measurement of spliced, unspliced, and total *XBP1* mRNA levels, expressed as fold changes relative to the levels in cells infected with empty adenovirus. F) ER stress markers analysis. Left panel: A representative Western blot analysis of ER stress markers BIP and CHOP in non‐infected cells (empty virus) and infected wild‐type and *SEC16A* mutant cells. Right panel: Quantification of BIP and CHOP in wild‐type and mutant HEK293T cell lines with exogenous amylase expression, compared to wild‐type cells with no exogenous amylase expression (empty virus control). G) UPR pathway marker analysis. Left panel: A representative Western blot analysis of UPR pathway markers PERK, ATF6, and IRE1 in non‐infected cells (empty virus) and infected wild‐type and *SEC16A* mutant cells. Right panel: Quantification of UPR pathway markers, compared to empty virus control. FC denotes fold change. All experiments were conducted using cell lines homozygous for the respective variants. For panels showing quantitative data, values represent mean ± SD from three independent experiments. Statistical significance indicated as n.s. (not significant) and ^**^ (*p* < 0.01), determined using one‐way ANOVA with Tukey's multiple comparisons test.

The selective export of proteins and lipids from the ER is mediated by COPII, which assembles at discrete sites on the membrane known as ER exit sites (ERESs).^[^ ^24^ ^]^ COPII consists of two proteinaceous layers: the inner layer (a flexible SEC23‐SEC24 lattice) and the outer layer (SEC13‐SEC31A heterotetramers). Since Sec16A orchestrates the formation of COPII‐coated vesicles by directly interacting with several components of COPII, including SEC31A,^[^ ^24^ ^]^ we postulated that p.Arg1495Gln and p.Val1947Leu would affect the formation of COPII vesicles. This hypothesis was substantiated by staining *AMY2A*‐virus infected cell lines for SEC16A and SEC31A. In the p.Arg1495Gln and p.Val1947Leu cell lines, a marked reduction in the number of ERESs was observed compared to the wild‐type and p.Arg1039Cys HEK293 cells (**Figure** **3**A). Although we prefer the explanation that the reduced immunofluorescent signals of SEC31A are due to reduced COPII assembly, we cannot exclude the possibility that the reduced signals may also be related to decreased expression of SEC31A.

The secretion defect in the p.Arg1495Gln and p.Val1947Leu cell lines suggested the possibility of ER stress due to protein overload.^[^ ^25^, ^26^ ^]^ To investigate this, we first tracked the intracellular fate of exogenously expressed CPA1 protein. After inhibiting protein synthesis with cycloheximide, a higher accumulation of CPA1 protein was noted in the p.Arg1495Gln and p.Val1947Leu cell lines compared to the wild‐type and p.Arg1039Cys HEK293 cell lines (Figure 3B,C), indicating impaired protein secretion and consequent protein overload.

Next, we examined *XBP1* mRNA splicing, a key ER stress marker, in the different cell lines under exogenous zymogen expression. The spliced/unspliced *XBP1* ratio was substantially higher in the p.Arg1495Gln and p.Val1947Leu cells than in the wild‐type and p.Arg1039Cys cells, while total X‐box‐binding protein 1 (*XBP1*) mRNA levels were comparable across these cell lines (Figure 3D,E). Additionally, we analyzed two other ER stress markers, immunoglobulin heavy chain binding protein (BIP) and C/EBP homologous protein (CHOP). Both markers were upregulated in the p.Arg1495Gln and p.Val1947Leu cell lines, but not in the Arg1039Cys cell line (Figure 3F).

Finally, we explored unfolded protein response (UPR) activation by detecting three main pathways: PRKR‐like ER kinase (PERK), activating transcription factor 6 (ATF6), and inositol‐requiring protein 1 (IRE1). All three markers were significantly upregulated in p.Arg1495Gln and p.Val1947Leu cell lines (Figure 3G).

Taken together, these results serve to confirm our hypothesis by demonstrating that the p.Arg1495Gln and p.Val1947Leu variants disrupt the secretory pathway, leading to protein accumulation and subsequent cellular stress.

### 2.7 Impaired Zymogen Secretion and Exacerbated ER Stress in Primary Pancreatic Acini of *Sec16a^+/−^* Mice

**Figure 4:**
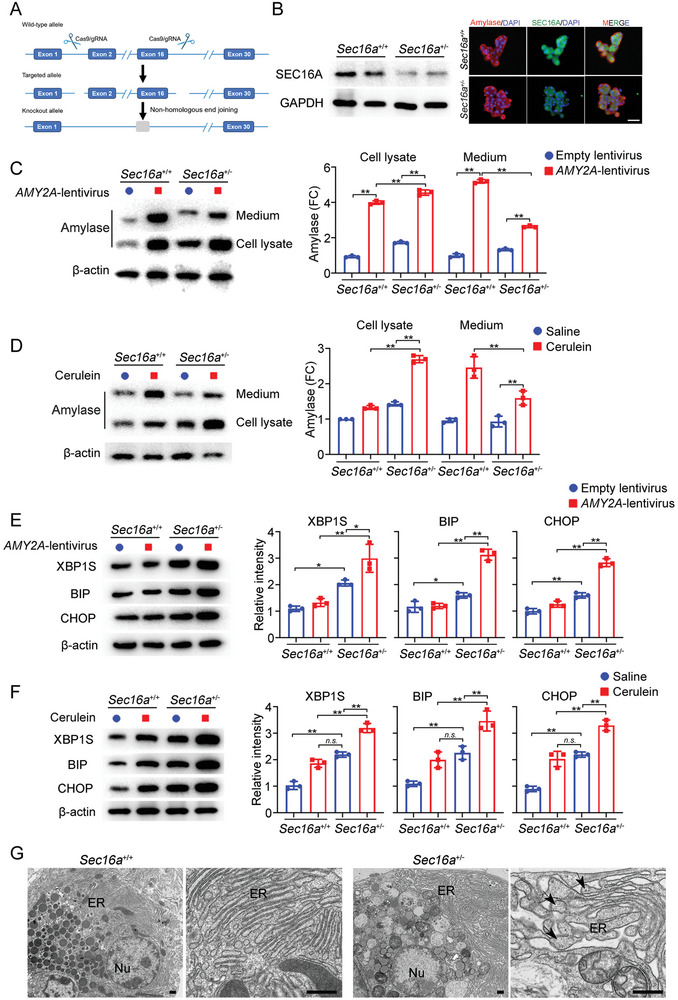
Impact of *Sec16a* knockout on protein secretion and ER stress in primary pancreatic acini. A) Schematic illustrating the generation of *Sec16a* knockout mice using CRISPR/Cas9‐mediated non‐homologous end joining. B) Left panel: Western blot analysis of pancreatic tissues from *Sec16a* ^*+/+*^ and *Sec16a* ^*+/−*^ mice. Right panel: Immunofluorescence analysis of *Sec16A* protein levels (green fluorescence), co‐stained with amylase (acinar marker, red fluorescence) in isolated pancreatic acini from 8‐week‐old *Sec16a^+/+^* and *Sec16a^+/−^* mice. Scale bar: 50 µm. C) Left panel: Immunoblots showing amylase levels in lysates and media from cultured pancreatic acinar cells isolated from *Sec16a^+/+^* and *Sec16a^+/−^* mice, infected with either *AMY2A*‐lentivirus or empty lentivirus. Right panel: Graph of amylase expression normalized to actin in the *AMY2A‐*lentivirus infected group compared to the control group. FC, fold change. D) Left panel: Immunoblots showing amylase levels in lysates and media from cultured pancreatic acinar cells isolated from *Sec16a^+/+^* and *Sec16a^+/−^* mice treated with cerulein or saline. Right panel: Graph illustrating amylase levels, normalized to actin, in the cerulein‐treated group relative to the control group. FC, fold change. E) Expression of ER stress markers (*XBP1* [spliced], BIP, and CHOP) in cultured pancreatic acinar cells with/without *AMY2A‐*lentivirus infection. Left: Representative immunoblots. Right: Quantitative analysis. F) Left panel: Immunoblots of ER stress markers (*XBP1* [spliced], BIP, and CHOP) in cultured pancreatic acinar cells with/without cerulein stimulation. Right panel: Relative intensity graph. G) Transmission electron microscopy (TEM) images showing ultrastructure changes in isolated pancreatic acinar cells of *Sec16a^+/+^* and *Sec16a^+/−^* mice. Arrows highlight unfolded protein accumulation in the ER. Scale bar: 1 µm. For panels with quantitative analysis, data are presented as mean ± SD. Statistical significance determined using two‐way ANOVA with Tukey's test: n.s., not significant; ^*^, *p* < 0.05; ^**^, *p* < 0.01.

To validate the above in vitro findings, we generated *Sec16a* knockout mice using CRISPR/Cas9 gene editing (**Figure** **4**A). Homozygous *Sec16a* knockout resulted in embryonic lethality, whereas heterozygous mice (*Sec16a^+/−^*) exhibited normal lifespan and development. Histological examination of key organs (pancreas, liver, heart, kidneys, lungs) under a light microscope showed no obvious abnormalities in adult mice under natural growth conditions. Consequently, all subsequent experiments were conducted using heterozygous knockout mice.

As shown in Figure 4B, *Sec16a^+/−^* mice displayed significantly reduced SEC16A expression compared to their wild‐type counterparts. We first explored the effects of heterozygous *Sec16a* knockout on cellular secretion capabilities using primary pancreatic acini as a model system. Pancreatic acinar cells were isolated from 8‐week‐old *Sec16a^+/−^* and *Sec16a^+/+^* mice, cultured for 24 h, and then divided into two experimental groups.

In the first experiment, we mimicked the impact of overexpressed secretory proteins on ER secretion by infecting pancreatic acini with *AMY2A*‐lentivirus. In the second experiment, we stimulated the pancreatic acini with cerulein to establish a cell‐culture model of pancreatitis. For the *AMY2A*‐lentivirus group, cells were infected with empty or *AMY2A*‐lentivirus for 24 h before collecting cell lysates and media. For the cerulein group, cells were treated with 100 nm cerulein or saline for 1 h, followed by an additional hour in a fresh medium.

Western blot analysis from both experiments showed significantly lower amylase secretion in pancreatic acinar cells of *Sec16a^+/−^* mice compared to *Sec16a^+/+^* mice (Figure 4C for *AMY2A*‐lentivirus infection; Figure 4D for cerulein stimulation). Analysis of ER stress makers revealed higher ER stress levels in pancreatic acinar cells of *Sec16a^+/−^* mice compared to *Sec16a^+/+^* mice, even without viral infection or cerulein stimulation (Figure 4F, blue dots). Additionally, ER stress was further exacerbated in the pancreatic acinar cells of *Sec16a^+/−^* mice relative to *Sec16a^+/+^* mice after *AMY2A*‐lentivirus transfection (Figure 4E, red squares) or cerulein‐stimulation (Figure 4F, red squares).

Finally, TEM evaluation of isolated pancreatic cells (without viral infection or cerulein stimulation) from *Sec16a^+/−^* mice showed increased intracellular vacuolization and visible ER dilations with protein overload compared to *Sec16a^+/+^* mice (Figure 4G).

These results collectively demonstrate that heterozygous loss of *Sec16a* function leads to impaired zymogen secretion and exacerbated ER stress, mirroring the protein overload‐induced cellular stress observed in our variant knock‐in cell studies.

### 2.8 *Sec16a^+/−^* Mice Exhibit Exacerbated Cerulein‐Induced Acute Pancreatitis (AP) and CP

**Figure 5:**
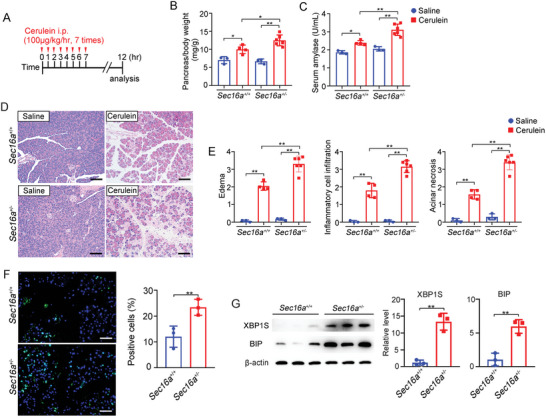
Genetic knockdown of *Sec16a* exacerbates cerulein‐induced AP. A) Schematic of the cerulein‐induced AP protocol. B) Comparison of pancreas/body weight ratios in *Sec16a^+/+^* and *Sec16a^+/−^* mice following cerulein‐induced AP. C) Serum amylase levels (U/mL) in *Sec16a^+/+^* and *Sec16a^+/−^* mice treated with cerulein or saline. D) H&E‐stained pancreas sections from *Sec16a^+/+^* and *Sec16a^+/−^* mice treated with cerulin or saline. Scale bar: 100 µm. E) Histology scores of AP in *Sec16a^+/+^* and *Sec16a^+/−^* mice. F) Left panel: TUNEL staining (green signal) illustrating apoptosis in *Sec16a^+/+^* and *Sec16a^+/−^* mice, with nuclei counterstained using DAPI. Scale bar: 100 µm. Right panel: Quantitative analysis of apoptosis‐positive cells. G) Expression of ER stress markers (XBP1 [spliced] and BIP) in the cerulein‐induced AP model of *Sec16a^+/+^* and *Sec16a^+/−^* mice (*n* = 3). Left: Representative immunoblots. Right: Quantitative analysis. For panels with quantitative analysis, data are presented as mean ± SD (*n* = 3–6). Statistical significance was assessed using a two‐tailed unpaired Student's *t*‐test for column analysis and two‐way ANOVA with Tukey's test for grouped analysis: ^*^, *p* < 0.05; ^**^, *p* < 0.01.

Next, we investigated whether heterozygous knockout of *Sec16a* affects the severity of pancreatitis in the context of cerulein‐induced AP. Using parameters such as pancreas mass, plasma amylase levels, and histological changes of pancreatic sections stained with hematoxylin‐eosin, we assessed the severity of AP in 8‐week‐old *Sec16a^+/+^* and *Sec16a* ^+/−^ mice following cerulein stimulation (**Figure** **5**A). While normal 8‐week‐old *Sec16a^+/+^* and *Sec16a* ^+/−^ mice showed no significant pancreatic differences (Figure S3, Supporting Information), cerulein‐treated *Sec16a^±^* mice demonstrated significantly increased severity of pancreatitis compared to cerulein‐treated wild‐type mice. This was evidenced by an elevated pancreas/body weight ratio, higher serum amylase levels, and more severe histology scores (Figure 5B–E; Figure S4A, Supporting Information). Although intrapancreatic trypsin activity showed no significant differences between *Sec16a* ^+/−^ and *Sec16a^+/+^* mice in cerulein‐induced AP model (Figure S4B, Supporting Information), massive infiltration of inflammatory cells was observed in *Sec16a* ^+/−^ mice post‐cerulein treatment (Figure S4C,D, Supporting Information). Additionally, terminal transferase‐mediated dUTP nick end‐labeling (TUNEL) staining and elevated expression of BIP and spliced XBP1 revealed increased acinar cell apoptosis and ER stress in *Sec16a* ^+/−^ mice compared to *Sec16a^+/+^* mice (Figure 5F,G). Altogether, these data demonstrated that the heterozygous knockout of *Sec16a* aggravates the severity of cerulein‐induced AP.

**Figure 6:**
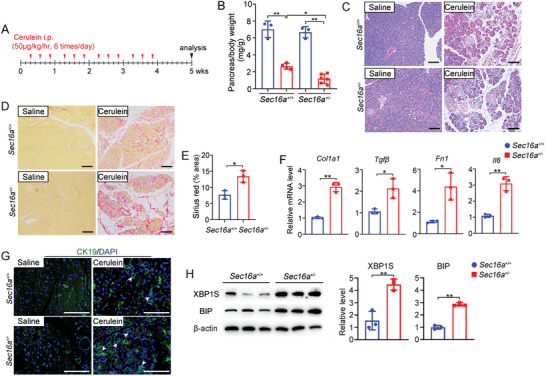
Enhanced severity of cerulein‐induced CP in *Sec16a* ^*+/−*^ mice. A) Schematic of the cerulein‐induced CP protocol. B) Pancreas/body weight ratios in *Sec16a^+/+^* and *Sec16a^+/−^* mice after cerulein‐induced CP. C) Representative H&E‐stained pancreas sections. Scale bar: 100 µm. D) Sirius red staining of pancreas sections from *Sec16a^+/+^* and *Sec16a^+/−^* mice. Scale bar: 100 µm. E) Quantification of positive‐staining areas using ImageJ software. F) Quantification of expression levels of fibrogenic factor genes (*Col1a1*, *Tgfβ*, and *Fn1*) and the proinflammatory factor gene interleukin‐6 (*Il6*) by quantitative RT‐PCR. G) CK‐19 immunofluorescence staining to evaluate acinar‐to‐ductal metaplasia in pancreata, with nuclei stained blue by DAPI. Scale bar: 100 µm. H) Expression of ER stress markers (XBP1 [spliced] and BIP) in cerulein‐induced CP model of *Sec16a^+/+^* and *Sec16a^+/−^* mice (*n* = 3). Left: Representative immunoblots. Right: Quantitative analysis. All data represent mean ± SD (*n* = 3–6). ^*^, *p* < 0.05; ^**^, *p* < 0.01. Two‐tailed unpaired Student's *t*‐test for column analysis; two‐way ANOVA with Tukey's test for grouped analysis.

Finally, we evaluated the impact of heterozygous knockout of *Sec16a* in a CP model (**Figure** **6**A). Cerulein‐treated *Sec16a* ^+/−^ mice exhibited a notable reduction in pancreas mass compared to *Sec16a^+/+^* mice (Figure 6B; Figure S5A, Supporting Information). Immunohistochemistry‐stained acinar amylase was significantly lower in *Sec16a^+/−^* mice, reflecting severe atrophy of the exocrine pancreas (Figure S5B, Supporting Information). Hematoxylin and eosin (H&E) staining of pancreas sections from *Sec16a^+/−^* mice showed pronounced acinar cell atrophy (Figure 6C). Sirius red and Masson's trichrome staining, along with immunohistochemistry of the fibrogenic marker α‐SMA, indicated higher levels of collagen deposition and fibrosis in *Sec16a^+/−^* mice than in *Sec16a^+/+^* mice (Figure 6D,E; Figure S5C, Supporting Information). Gene expression analyses revealed significant upregulation of three fibrogenic factor genes (i.e., collagen type I alpha 1 [*Col1a1*], fibronectin [*Fn1*], and transforming growth factor‐β [*Tgf‐β*]) and one proinflammatory factor gene (i.e., interleukin‐6 [*Il6*]) in the pancreata of *Sec16a^+/−^* mice (Figure 6F). Cerulein‐induced pancreatitis in *Sec16a^+/−^* mice was characterized by an increased infiltration of inflammatory cells, including CD45 leukocyte and F4/80 macrophages (Figure S5D, Supporting Information). Staining for the ductal cell marker cytokeratin 19 (CK19) revealed increased proliferation of pancreatic duct cells in pancreas sections from *Sec16a^+/−^* mice, implying acinar‐to‐ductal metaplasia instead of acinar cell dropout (Figure 6G). Significantly elevated ER stress markers were also observed in the *Sec16a^+/−^* mice compared to the *Sec16a^+/+^* mice (Figure 6H).

## 3 Discussion

The genetic landscape of CP is mainly characterized by genes expressed specifically or predominantly in pancreatic acinar cells, such as *PRSS1, SPINK1, CTRC, CPA1, CEL, CTRB1‐CTRB2*, and *PNLIP*,^[^ ^3^, ^6^, ^7^, ^8^, ^9^, ^10^, ^11^ ^]^ or pancreatic ductal cells, notably *CFTR*.^[^ ^17^, ^18^ ^]^ By contrast, the recently reported CP susceptibility gene, *TRPV6*,^[^ ^15^, ^16^ ^]^ is expressed in multiple tissues, among which pancreas ranks second in terms of *TRPV6* RNA expression according to the Human Protein Atlas database.^[^ ^27^ ^]^

In this study, we found a significant association between rare *SEC16A* variants and CP. The association was significant across both Chinese and French cohorts, as demonstrated in an aggregate analysis of all rare variants and those with a CADD score of ≥20. This significant association was also observed when combining data from three biobanks. Notably, all four pLoF variants identified—one from trio exome sequencing and three from targeted sequencing—were found exclusively in patients, not in controls. Based upon gnomAD data, it may be concluded that pLoF variants in the *SEC16A* gene have been subject to moderate negative selection. Furthermore, in patients that did not carry a known pathogenic genotype involving *PRSS1*, *SPINK1*, *CTRC* and/or *CFTR*, the presence of rare *SEC16A* variants was significantly associated with an earlier onset of CP. These various lines of genetic evidence strongly support the hypothesis that loss‐of‐function variants in *SEC16A* are novel risk factors for CP.

SEC16A is a key regulator in the formation of COPII vesicles, which mediate protein transport from the ER to the Golgi apparatus. SEC16A interacts with key components of the COPII machinery, regulating their function.^[^ ^23^ ^]^ Depletion of SEC16A reduces the number of COPII budding sites on the ER, thereby slowing the rate at which secretory cargoes exit the ER and disrupting the early secretory pathway.^[^ ^24^, ^28^ ^]^ These observations align with those from *Sec23b* (encoding another key component of COPII) conventional or conditional knockout mouse models. These models exhibited a decrease in zymogen granules, ER distention, and increased apoptosis in the pancreatic acini, leading to pancreatic degeneration.^[^ ^29^, ^30^, ^31^ ^]^ Additionally, *Sec23b* hemizygosity (*Sec23b^ki/ko^*) mice (ki, knocked in p.E109K mutation—the most common human mutation in congenital dyserythropoietic anemia type II patients; ko, knockout) displayed phenotypes consistent with CP.^[^ ^32^ ^]^ These findings underscore the critical role of ER‐to‐Golgi transport in the pathogenesis of pancreatitis. Unlike previously identified CP susceptibility genes, *SEC16A* is ubiquitously expressed, with high abundance in the pancreas.^[^ ^33^ ^]^ SEC16A's role has been rarely studied in specific tissues or organs, but it has been reported to be involved in hepatic lipid metabolism,^[^ ^34^ ^]^ whole‐body glucose homeostasis,^[^ ^35^ ^]^ and both conventional and unconventional secretion of CFTR.^[^ ^36^ ^]^

Given SEC16A's crucial role and the distinct mechanism of ER stress resulting from misfolding variants underlying CP,^[^ ^5^ ^]^ we first conducted functional analyses of selected *SEC16A* variants using CRISPR/Cas9‐edited HEK293T cell lines.^[^ ^37^ ^]^ We focused on two rare missense variants enriched in Chinese patients (p.Arg1495Gln and p.Val1947Leu) and one common variant equally present in patients and controls (p.Arg1039Cys). Our experiments in the variant knock‐in cell model demonstrated that loss‐of‐function variants in SEC16A impede pancreatic enzyme secretion, ultimately leading to protein accumulation and inducing ER stress. More specifically, the results suggest that the excessive accumulation of proenzymes in the ER triggers the ER overload response (EOR), inducing ER stress and leading to the inflammatory response.^[^ ^38^, ^39^ ^]^ Additionally, we found that the UPR pathways (encompassing PERK, ATF6, and IRE1) were concurrently activated, often co‐occurring with EOR.^[^ ^40^ ^]^ Whereas the UPR facilitates cellular adaptation to ER stress, persistent UPR activation leads to cell death. Furthermore, PERK activation also upregulates genes involved in autophagy.^[^ ^41^ ^]^ These pathological mechanisms may contribute to the progression of pancreatitis.

Two points are worth mentioning in relation to the use of the variant knock‐in cell model. First, given the study's relevance to pancreatitis, using pancreatic acinar cells would be ideal. However, no human acinar cell lines are available, and the widely used rat acinar cell line AR42J is a doubtful alternative for this purpose owing to its diminished secretory function.^[^ ^42^ ^]^ Instead, HEK293 or HEK293T cells have been used effectively to study the secretory effects of missense variants in CP genes such as *PRSS1*,^[^ ^43^ ^]^ *SPINK1*,^[^ ^42^, ^44^ ^]^ *CTRC*,^[^ ^45^ ^]^ *CPA1*,^[^ ^46^ ^]^ *CEL*,^[^ ^9^, ^47^ ^]^ and *PNLIP*,^[^ ^11^, ^48^ ^]^ as well as the function of SEC16A.^[^ ^28^, ^36^, ^49^, ^50^ ^]^ Second, to minimize interference from endogenous SEC16A, we conducted functional analyses on *SEC16A* homozygous missense variants in CRISPR/Cas9‐edited HEK293T cell lines. This approach is analogous to transfecting cell lines that do not express the gene of interest with plasmids harboring missense variants (e.g., the functional effects of missense variants in the aforementioned CP genes were analyzed this way). One *caveat* of this approach is the inability to analyze potential dominant negative effects of the studied missense variant. However, this limitation does not affect the main conclusion of the study.

Findings from the variant knock‐in cell model were further validated using *Sec16a^+/−^* mice, which exhibited more severe pancreatitis under cerulein stimulation compared to their wild‐type counterparts. Cerulein, an analog of cholecystokinin, triggers excessive secretion of pancreatic enzymes and enhances the exocytosis of zymogen granules from acinar cells,^[^ ^51^ ^]^ thereby increasing the ER's burden for protein modification and secretion. Considering the vital role of SEC16A in ER‐to‐Golgi trafficking, its reduced expression in *Sec16a^+/−^* mice likely aggravates the effects of cerulein stimulation. In short, our mouse studies confirmed that the loss of SEC16A function increases susceptibility to severe and progressive pancreatitis by impairing ER‐to‐Golgi transport and inducing ER stress. Notably, this pathogenic mechanism aligns with findings from other studies. For example, impaired ER‐to‐Golgi protein trafficking contributes to ER stress in lipotoxic mouse beta cells,^[^ ^52^ ^]^ and recessive mutations in *YIPF5* disrupt ER‐to‐Golgi trafficking, causing neonatal diabetes and microcephaly through ER stress.^[^ ^53^ ^]^

The origins of ER stress‐related mechanisms described in this study differ fundamentally from those previously reported in pancreatic pathology. In our study, ER stress results from the reduced secretion of normally synthesized pancreatic zymogens due to impaired ER‐to‐Golgi trafficking caused by reduced SEC16A function. By contrast, previously described mechanisms involve misfolded zymogens attributable to missense variants.^[^ ^5^ ^]^ Notably, misfolding variants in *CPA1* ^[^ ^54^, ^55^ ^]^ and *PRSS1* ^[^ ^56^ ^]^ are often associated with hereditary pancreatitis, characterized by large effect sizes and a frequent family history. In this study, we identified four clear loss‐of‐function *SEC16A* variants (c.6491_6492delTG, c.3803‐1G>A, c.6691delA, and c.3493_3513del). All four variants were found exclusively in patients without a family history (note, however, that the c.6491_6492delTG variant occurred *de novo*). Furthermore, the overall ORs from the analysis of all rare *SEC16A* variants across all populations are relatively small (Figure 1A). These differences in genetic effects may be intricately related to the underlying pathogenic mechanisms.

Another notable finding is that there were no significant differences in intrapancreatic trypsin activity between *Sec16a^+/−^* and *Sec16a^+/+^* mice after cerulein stimulation (Figure S4B, Supporting Information). This phenomenon may be explained as follows: pathological trypsinogen activation mainly occurs with the co‐localized lysosomal and digestive enzymes,^[^ ^57^ ^]^ whereas no trypsinogen activation occurs during ER synthesis or in the ER‐to‐Golgi transport pathway, possibly due to the co‐synthesis of trypsinogen with pancreatic secretory trypsin inhibitor.^[^ ^58^ ^]^ Our results suggest that ER stress induced by protein overload is independent of the pathological intrapancreatic activation of trypsinogen in pancreatitis development.

As previously noted, *SEC16A* is a ubiquitously expressed gene, with the pancreas being one of the organs with the highest expressing levels.^[^ ^33^ ^]^ To date, only a few studies have suggested potential links between *SEC16A* variants and human diseases. One such case involved a three‐amino‐acid in‐frame deletion in *SEC16A* (p.Ser369_Ala371del) in a well‐characterized, multigenerational family with axial spondylarthritis. However, this association was complicated by a co‐inherited, unambiguous loss‐of‐function variant in the *MAMDC4* gene.^[^ ^59^ ^]^ Similarly, the significance of three rare *SEC16A* missense variants (c.1019G>A [p.Gly340Glu], c.1037G>A [p.Arg346His] and c.1480G>C [p.Gly494Arg]) identified in pediatric inflammatory bowel disease patients remains uncertain due to the absence of functional data and their co‐inheritance with rare variants in other candidate genes.^[^ ^60^ ^]^ Additionally, *SEC16A* c.3820C>T (p.Arg1274Cys) was identified as a candidate variant associated with various developmental delays and abnormalities in a Saudi Arabian girl.^[^ ^61^ ^]^ In short, the pathological relevance of these associations remains to be fully established.

Taking these findings^[^ ^59^, ^60^, ^61^ ^]^ into consideration, we re‐examined our cohort of 1061 Chinese CP patients for the potential presence of extra‐pancreatic chronic inflammatory syndromes. However, we were unable to draw any meaningful conclusions about other potential disease associations owing to the rare occurrences of such incidences (e.g., chronic bronchitis and epididymitis in isolated cases). Whilst future research may uncover associations between *SEC16A* variants and extra‐pancreatic pathologies, it is crucial to recognize the unique vulnerability of pancreatic acinar cells. These cells synthesize and secrete abundant proteins and have a substantially large volume of ER,^[^ ^62^ ^]^ rendering them especially susceptible to disruptions in SEC16A function and ER homeostasis. Nevertheless, a global gene knockout in mice may impact other organs, and future studies could benefit from creating a pancreas‐specific knockout mouse model.

In summary, this study is the first to identify an association between rare *SEC16A* variants and CP and to construct a *Sec16a* knockout mouse model. Our functional analyses in cellular and mouse models show that loss‐of‐function variants in *SEC16A* disrupt ER‐to‐Golgi trafficking, leading to protein overload‐induced ER stress. These findings implicate a role for SEC16A in CP pathogenesis and highlight the need for ongoing research into the genetic underpinnings of this complex disease.

## 4 Experimental Section

### Chinese CP Discovery Cohort

Sixteen teenage CP patients along with their healthy parents were subjected to exome sequencing. These patients had remained genetically unexplained after a comprehensive screen for mutations in their *PRSS1*, *SPINK1*, *CFTR*, *CTRC*, and *CLDN2* genes.^[^ ^63^ ^]^ The 1061 Chinese CP patients and 1196 Chinese healthy controls (584 males and 612 females) subjected to targeted sequencing of the *SEC16A* gene have been described elsewhere.^[^ ^22^ ^]^

### French Replication Cohort

The cohort comprised 383 CP patients, split between 192 with idiopathic CP and 191 with familial or hereditary CP (for term definitions, see Masson et al.^[^ ^64^ ^]^). These patients were characterized by the absence of known causative mutations in *PRSS1*, *SPINK1*, and *TRPV6* genes, as well as the lack of *CFTR* genotypes comprising a severe allele plus a mild allele. The 574 subjects in the FREX Project (http://lysine.univ‐brest.fr/FrExAC/) were used as controls.

Ethical approval for this study was obtained from the Institutional Ethics Committees of both the Shanghai (China) groups and Brest (France). Informed consent was obtained from all participants. In cases involving participants under the age of 18, consent was appropriately acquired from their parents or legal guardian.

### Biobank Data

A single‐variant association data from three biobanks were collected: i) FinnGen (freeze 9, April 2022):^[^ ^65^ ^]^ Data with Risteys for CP (K11_CHRONPANC), including 3320 CP patients and 330903 controls; ii) UK Biobank (pan‐UKB, version 0.4) [Pan‐UKB team. https://pan.ukbb.broadinstitute.org. 2020]: Data with International Classification of Diseases (ICD)−10 entries for CP (K86), comprising 607 CP patients and 417106 controls; iii) The BioBank Japan Project:^[^ ^66^ ^]^ Data with ICD‐10 entries for CP, including 457 CP patients and 177471 controls.

### Variant Nomenclature and Reference Sequences

Variant nomenclature adhered to the guidelines set by the Human Genome Variation Society (HGVS).^[^ ^67^ ^]^ NM_01 4866.2 was used as the *SEC16A* mRNA reference sequence. The genomic sequence of *SEC16A* was sourced from the human GRCh37/hg19 build, accessible via the UCSC Genome Browser (https://genome.ucsc.edu/).

### Exome Sequencing and Targeted Sequencing, Variant Identification, and Validation

Genomic DNA from the 16 Chinese trios was processed to create paired‐end read libraries with a 100‐bp fragment size, following the protocols specified by Illumina (Illumina, California). Exome sequencing was carried out as previously described.^[^ ^68^ ^]^ Candidate variants were identified from VCF (Variant Call Format) files based on the following criteria: i) a minimum read depth of 10 in the trios, ii) inclusion of only missense, nonsense, small indel or canonical splice site variants, and iii) a minor allele frequency of <1% in the ExAC database. For targeted sequencing, a total of 62 specific primer pairs were designed (Table S4, Supporting Information) for the coding sequence and exon/intron boundaries of the *SEC16A* gene using Primer3 (http://primer3.org). All called rare variants were subjected to validation by Sanger sequencing (Table S5, Supporting Information). For further details, see the Supporting Information.

### *SEC16A* Variants in Biobanks

The *SEC16A* variants identified in the CP patients and controls from the FinnGen, UK Biobank, and BioBank Japan datasets were evaluated using a gene‐based burden analysis. The inclusion criteria for variants in this analysis were: i) missense, nonsense, small indel, or canonical splice site variants; ii) variants with a minor allele frequency of <1% in the gnomAD database. The frequencies of these aggregated variants in patients versus controls using the *Chi*‐square test were compared. Additionally, a meta‐analysis was performed using a fixed‐effects model, implemented with the meta package (version 6.5) in R.

### In Silico Prediction of Pathogenicity

To predict the pathogenicity of the identified variants, The CADD method was utilized.^[^ ^69^ ^]^ This tool effectively measures the deleteriousness of genetic variants and aids in prioritizing potential causal variants in genetic analyses. It is accessible through the CADD website (GRCh37‐v1.6; 2019. https://cadd.gs.washington.edu). Variants yielding a PHRED‐like CADD‐score of ≥20 were classified as likely pathogenic.

### Generation of Mutant Cell Lines Via the CRISPR/Cas9 System

HEK293T cell lines carrying the c.3115C>T (p.Arg1039Cys), c.4484G>A (p.Arg1495Gln), and c.5839G>T (p.Val1947Leu) knock‐in alleles of the *SEC16A* gene using the CRISPR/Cas9 system, as per previously established methods were generated.^[^ ^37^, ^70^ ^]^ The experimental procedures encompassed several key steps: i) construction of a lentiviral Cas9 vector containing single‐guide RNA (sgRNA) (Table S6, Supporting Information), ii) validation of sgRNA activity, iii) creation of a corrective adeno‐associated virus containing the donor template (single‐stranded oligodeoxynucleotides), iv) co‐transfection of Cas9, sgRNA, and the donor template into HEK293T cells, and v) screening of CRISPR/Cas9 clones for mutations. These procedures were carried out by Obio Technology (Shanghai, China). The validation of clones was performed by polymerase chain reaction (PCR) and Sanger sequencing by the Shanghai participating group (Figure S2, Supporting Information).

### Cell Culture and Transfection Using Lentiviral Infection

Wild‐type and mutant HEK293T cells were cultured in Dulbecco's Modified Eagle Medium (DMEM) supplemented with 10% fetal bovine serum and 1% penicillin/streptomycin (Invitrogen). Lentivirus packed with either *AMY2A* (NM_000699.4), *PRSS1* (NM_0 02769.5) or *CPA1* (NM_0 01868.4) were purchased from Obio Technology (Shanghai, China). For lentiviral infection, HEK293T cells were initially seeded in 6‐well plates at a density of 5 × 10^4^ cells per well for 24–36 h until they reached 50–70% confluence. Subsequently, the culture medium was replaced with 100 µL of either lentivirus‐*AMY2A*, lentivirus‐*PRSS1*, or lentivirus‐*CPA1*, each with a titer of 1 × 10^8^ transducing units per mL (TU/mL) in fresh media. The cells were incubated with the virus for 24 h, after which the virus‐containing medium was discarded and replaced with fresh normal medium. Post‐infection, cells that successfully integrated the virus were selected using 10 µg mL^−1^ puromycin for a minimum of 72 h. These puromycin‐selected cells were then utilized for subsequent experiments.

### Immunocytochemistry and ERES Quantification

Initially, cells were fixed with 4% paraformaldehyde for 10 min. These were then permeabilized and blocked with a blocking buffer composed of PBS with 0.1% Triton X‐100 and 1% BSA for 30 min. Subsequently, the cells were incubated overnight at 4 °C with primary antibodies (anti‐SEC16A and anti‐SEC31A, both at a 1:200 dilution). This step was followed by a 30 min incubation at 37 °C with fluorescence‐conjugated secondary antibodies. The nuclei of the cells were stained using 4′, 6‐diamidino‐2‐phenylindole (DAPI) procured from Sigma–Aldrich (St. Louis, MO). The captured images were processed using ImageJ software. For the quantification of SEC31‐positive ERES structures, images were acquired using a LeicaSP5 confocal microscope and THUNDER imagers on a Mica wide‐field microscope. The quantification was then performed using the particle count tool in ImageJ, as detailed in a previously study.^[^ ^28^ ^]^

### Rate of Cellular Secretory Protein Clearance

To assess the rate of clearance of cellular secretory proteins, an inhibition of protein synthesis was performed. Cells were initially cultured in 24‐well plates at a density of 1 × 10^5^ cells per well for 24 h. Subsequently, the cells were treated with cycloheximide at a concentration of 100 µg mL^−1^. Following a 120 min treatment period, the cells were fixed using 3% paraformaldehyde for 15 min. The subsequent immunofluorescence procedures involved staining the secretory proteins using an anti‐V5 tag antibody (13202, Cell Signaling Technology; 1:5000 dilution) and marking the ER using anti‐GRP94 (ab238126, Abcam; 1:200 dilution). Additionally, the cell nuclei were stained with DAPI. Images were acquired using a Leica SP5 confocal microscope and THUNDER imagers on a Mica wide‐field microscope.

### Generation of Sec16a Gene Knockout Mouse Model and Genotyping

All mouse experiments conformed to ethical guidelines and were approved by the Institutional Animal Care and Use Committee of Shanghai Changhai Hospital (CHEC[A.E]2022‐008). Mice on a C57BL/6J genetic background to generate the *Sec16a* knockout model was used, designed, and developed by Shanghai Model Organisms Center, Inc (Shanghai, China). The genomic DNA sequence of the *Sec16a* gene was retrieved from the Ensembl database (http://asia.ensembl.org).

Cas9 mRNA was transcribed in vitro with the mMESSAGE mMACHINE T7 Ultra Kit (Ambion, TX, USA) according to the manufacturer's instructions. Two guide RNAs (sgRNA1 targeted to intron 1, 5′‐AAGGAGATGCTGCTTATCAA‐3′; sgRNA2 targeted to intron 16, 5′‐TCACCAGTGCTTGCTTGTTA‐3′) of the *Sec16a* gene were transcribed in vitro using the MEGAshortscript Kit (ThermoFisher, USA). The Cas9 mRNA and sgRNAs were injected into zygotes of C57BL/6J mouse, resulting in the deletion of exons 2–16 of the *Sec16a* gene and subsequent fusion of the flanking introns through non‐homologous end joining. The zygotes were transferred to pseudopregnant recipients.

Genotyping involved analyzing DNA samples extracted from the tails or toes of F0 mice at 3 weeks of age, using a forward primer (5′‐CTGTCAGACTGGCAACAGGT‐3′) and a reverse primer (5′‐GACACCTGCACTCCCCTAAC‐3′). Positive F0 mice were selected and crossed with C57BL/6J mice to obtain F1 heterozygous *Sec16a* knockout mice. The genotype of F1 mice was identified by PCR and confirmed by sequencing. Male and female F1 heterozygous mice were intercrossed to produce homozygous *Sec16a* knockout mice. The mice were housed under a 12 h light‐dark cycle with ad libitum access to a standard diet. No phenotypic differences between the sexes were noted in the experiments.

### Isolation of Primary Pancreatic Acinar Cells

The isolation of primary pancreatic acinar cells was carried out following previously established protocols with some modifications.^[^ ^71^ ^]^ For detailed procedures, refer to the Supporting Information.

### Induction of Pancreatitis

The specific methodologies and protocols for developing both cerulein‐induced AP and CP mouse models are detailed in the Supporting Information.

### Histologic Analysis, Immunohistochemistry, Immunofluorescence, TUNEL Assay, TEM, and Measurement of Trypsin and Amylase Activity

Histological analysis was performed through the examination of five random fields at 100× magnification. Two independent investigators, blinded to the sample identities, graded the samples on a score from 0 to 4 for parameters such as edema, inflammatory cell infiltration, and acinar necrosis.^[^ ^72^ ^]^ The results were then quantified as means ± SEM. Comprehensive details regarding the antibodies used for immunohistochemistry and immunofluorescence, the TUNEL assay, the TEM procedure, and the protocols for measuring trypsin and amylase activity are provided in the Supporting Information.

### Reverse Transcription‐PCR and Quantitative PCR Analyses, Western Blot Analysis

For the semiquantitative measurement of *XBP1* mRNA splicing, PCR analyses were conducted using primers capable of amplifying both spliced and unspliced forms of *XBP1*, resulting in amplicons of 415‐bp and 441‐bp, respectively. Additionally, the primers used for genes encoding fibrogenic and proinflammatory factors, including *Col1a1*, *Fn1*, *Tgf‐β*, and *Il6*, are detailed in Table S7 (Supporting Information). Comprehensive information about the procedures and antibodies used for these analyses can be found in the Supporting Information.

### Statistical Analysis

The significance of differences in variant carrier and allele frequencies between patients and controls was assessed by a two‐tailed *Chi*‐square test or Fisher's exact test, utilizing GraphPad Prism software (version 8.0.1) for calculations. Meta‐analysis of all cohorts was conducted using R software (version 6.5), which included generating forest plots to visually represent the data. All experimental data are presented as means ± SD. A significant difference between control and multiple mutated groups was performed using one‐way or two‐way ANOVA analysis with Tukey's multiple comparisons test; a two‐tailed Student's *t*‐test was used for comparison between two groups. All experiments were performed at least three times. A *p‐*value <0.05 was considered to be statistically significant. The Kaplan–Meier method was used to plot survival curves; differences between the survival curves of groups were evaluated by a log‐rank test with a 0.05 significance level.

## Conflict of Interest

The authors declare no conflict of interest.

## Author Contributions

M.J.W., Y.C.W., and E.M. share co‐first authorship. M.J.W., Y.C.W., and E.M. performed the genetic and/or functional analysis, with substantial contributions from D.Y., X.Y.T., S.J.D., L.H.H., L.W. and L.J.W. S.J.D. and X.Y.T. performed the Sanger sequencing. Y.H.W. performed publicly databases analysis. Y.Y.Q. and W.B.Z. recorded the clinical data. M.J.W. and Y.C.W. performed animal experiments. M.J.W., Y.C.W., J.M.C., W.B.Z., and Z.L. wrote the manuscript. V.R. provided DNA samples and collected clinical data. D.N.C., C.F., and Z.S.L. critically revised the manuscript with important intellectual input. Z.S.L., W.B.Z., J.M.C. and Z.L. obtained the funding. W.B.Z., J.M.C., and Z.L. contributed to research design. All authors contributed to data interpretation and revision of the manuscript and approved the final version of the paper.

## Supporting information

Supporting Information

## Data Availability

The data that support the findings of this study are available from the corresponding author upon reasonable request.
