# Supplementary material for: SEC16A Variants Predispose to Chronic Pancreatitis by Impairing ER‐to‐Golgi Transport and Inducing ER Stress
Source: Adv Sci (Weinh). 2024 Aug 9;11(38):2402550. doi: 10.1002/advs.202402550 (PMC11481239; doi:10.1002/advs.202402550)
Supplement: Supplementary file 1 — Supporting Information [file ADVS-11-2402550-s001.docx]

Supporting Information

*SEC16A* Variants Predispose to Chronic Pancreatitis by Impairing ER-to-Golgi Transport and Inducing ER Stress

Min-Jun Wang,^#^ Yuan-Chen Wang,^#^ Emmanuelle Masson,^#^ Ya-Hui Wang, Dong Yu, Yang-Yang Qian, Xin-Ying Tang, Shun-Jiang Deng, Liang-Hao Hu, Lei Wang, Li-Juan Wang, Vinciane Rebours, David N. Cooper, Claude Férec, Zhao-Shen Li, Jian-Min Chen,* Wen-Bin Zou,* and Zhuan Liao*

M. J. Wang, Y. C. Wang, and E. Masson share co-first authorship.

*Correspondence Authors. Emails: liaozhuan@smmu.edu.cn; dr.wenbinzou@hotmail.com; jian-min.chen@univ-brest.fr

This file includes:

Supplementary Experimental Section

Table S1. Rare *SEC16A* nonsynonymous variants in the French CP cohort

Table S2. Rare *SEC16A* nonsynonymous variants in FREX

Table S3. Rare *SEC16A* nonsynonymous variants in three publicly available biobanks

Table S4. Primers for targeted next-generation sequencing of the *SEC16A* gene

Table S5. Primers for Sanger sequencing of the *SEC16A* gene

Table S6. Single guide RNA (sgRNA) sequences for the *CRISPR/Cas9* system

Table S7. Primers for quantitative reverse transcription PCR analysis of *Xbp1*, *Col1a1*, *Fn1*, *Il6*, and *Tgf-β* mRNAs

Figure S1. Meta-analysis of rare *SEC16A* variants with a CADD score of ≥20 in Chinese CP discovery cohort and replication cohorts

Figure S2. Sanger sequencing validation of three Cas9-edited HEK293T cell lines with indicated *SEC16A* mutations

Figure S3. Absence of pathological changes in *Sec16a^+/-^* mice 8 weeks after birth

Figure S4. Cerulein-induced AP in *Sec16a^+/+^* and *Sec16a^+/-^* mice

Figure S5. Cerulein-induced CP in *Sec16a^+/+^* and *Sec16a^+/-^* mice

**Supplementary Experimental Section**

*Exome sequencing and variant identification*: Genomic DNA libraries with 100-bp paired-end reads were prepared from 16 Chinese trios using protocols recommended by Illumina (Illumina, California). Exome sequencing was performed by our collaborators at the Bio-X Institutes of Shanghai Jiao Tong University, as described previously.^[1]^ Exome enrichment utilized the TruSeq Exome Enrichment kit (Illumina). Sequencing was performed on an Illumina HiSeq 2500 Genome Analyzer, yielding paired-end reads for each sample.

Candidate variant selection adhered to the following criteria: (i) a read depth greater than 10 in the trios; (ii) inclusion of missense, nonsense, small indel or canonical splice site variants; and (iii) a minor allele frequency below 1% in the ExAC database. All identified variants underwent confirmation via Sanger sequencing.

*Targeted sequencing of the SEC16A gene*: To analyze the entire coding sequence and exon/intron boundaries of the *SEC16A* gene in the Chinese population, we designed 62 target-specific primer pairs (**Table S1**) using Primer3 (http://primer3.org). The primers were synthesized with common adaptor sequences at their 5’ ends as previously described.^[2]^ The 62 primer pairs were divided into two multiplex primer pools. Pre-amplification of the tagged-gene amplicons, generation of a barcoded DNA library for multiplex high-throughput sequencing, quantification and clean-up of the DNA library and sequencing were performed essentially as previously described.^[2]^ Variant calling and filtering for point mutations, micro-insertions or micro-deletions were performed as previously described.^[3]^ The French patients were analyzed by targeted next-generation sequencing as previously described.^[4]^

*Variant validation by Sanger sequencing*: All called rare variants were subjected to validation by Sanger sequencing. Primer sequences used for amplifying target fragments are listed in **Table S2**. Polymerase chain reaction (PCR) was performed in 25 μl reaction mixtures containing 12.5 µL HotStarTaq Master Mix (Qiagen, Germany), 0.4 µM of each of the corresponding forward and reverse primers, and 1 μl DNA (concentration 10-50 ng/μL). The PCR program comprised an initial denaturation at 95°C for 15 minutes, followed by 30 cycles of denaturation at 95°C for 30 seconds, annealing at 56°C-64°C for 30 seconds, and extension at 72°C for 90 seconds, with a final extension step at 72°C for 10 minutes. PCR products were separated via agarose gel electrophoresis, and the bands of the correct size were excised and purified using a DNA Purification Kit (Tiangen, Beijing, China). Sequencing was performed using the BigDye Terminator Sequencing Kit (ABI) on an ABI 3730xl DNA analyzer. For variants identified in French patients, confirmation was achieved using Sanger sequencing on an 3130xl DNA analyzer.

*Isolation of primary pancreatic acinar cells*: Pancreatic acinar cells were isolated following a slightly modified version of a previously described method.^[5]^ Briefly, the pancreas was excised and perfused with 1% PBS. The tissue was then incubated with 2 mg/ml type Ⅳ collagenase (C5138, Sigma) for 20 minutes at 37℃. Following the enzymatic digestion, the tissue was mechanically disrupted, and the resulting pancreatic acinar cells were filtered through a 70 μm nylon cell strainer. The cells were then centrifuged at 1,000 rpm for 1 minute. The resulting cell pellet was resuspended and cultured in DMEM supplemented with 10% fetal bovine serum, 100 U/mL penicillin, and 100 mg/mL streptomycin for the specified durations. For the immunocytochemistry of isolated pancreatic acini, the cells were incubated overnight at 4°C with following primary antibodies: anti-amylase (66133-1-Ig, Proteintech; 1:200) and anti-mouse Sec16a (20025-1, Proteintech; 1:200). The following steps were as previously described.

*Induction of pancreatitis*: Acute pancreatitis (AP) model (**Figure 5A**): Mice underwent seven intraperitoneal injections of cerulein (100 μg/kg; HY-A0190, MedChemExpress), each administered at one-hour intervals, following 12 hours of fasting, with free access to water. Pancreatitis phenotypes peaked 12 hours after the first cerulein injection, at which point mice were humanely euthanized.^[6, 7]^

Chronic pancreatitis (CP) model (**Figure 6A**): The CP model involved six daily intraperitoneal injections of cerulein (50 μg/kg/hr), administered three days per week for four consecutive weeks. Mice were sacrificed seven days after the last injection.

Blood samples for measuring serum trypsin and amylase activity were obtained from the ophthalmic venous plexus. Pancreatic tissues were harvested and weighted to determine the pancreas/body weight ratio (mg/g), indicative of pancreatic edema or atrophy.

*Western blot analysis*: Cell lysates or 30 mg of pancreatic tissue were homogenized in 200-300 µL lysis buffer supplemented with the Halt^TM^ protease inhibitors cocktail. We loaded 50 µg homogenates onto 10% or 12% SDS-polyacrylamide gels for separation, followed by electroblotting onto polyvinylidene fluoride (PVDF) membranes (Merck Millipore). For protein secretion analysis, the volume of cell culture media used was equivalent to the corresponding lysate volume. The membranes were blocked with blocking buffer for 1 hour at room temperature, then incubated overnight at 4°C with primary antibodies: anti-amylase (3796, Cell Signaling Technology; 1:1000), anti-human SEC16A (PA5-52182, Invitrogen; 1:1000), anti-mouse Sec16a (20025-1, Proteintech; 1:1000), anti-V5 tag (13202, Cell Signaling Technology; 1:5000), anti-BIP (3177, Cell Signaling Technology; 1:1000), anti-CHOP (2895, Cell Signaling Technology; 1:1000), anti-spliced XBP1 (24868-1-AP, Proteintech; 1:1000), anti-PERK/EIF2AK3 (24390-1-AP, Proteintech; 1:1000), anti-ATF6 (24169-1-AP, Proteintech; 1:1000), anti-IRE1 (27528-1-AP, Proteintech; 1:1000), or anti-actin (ab8226, Abcam; 1:5000). Following three PBST washes, membranes were incubated with an HRP-conjugated secondary antibody at 37°C for 30 minutes. Protein bands were visualized using SuperSignal West Pico Chemiluminescent Substrate (Thermo Fisher, NY). Densitometry analysis of protein bands, performed using the ImageJ software (https://imagej.net/), was normalized to actin protein levels.

*Measurement of trypsin and amylase activity*: Cells were infected with lentivirus-*PRSS1* or lentivirus-*AMY2A* and re-plated into 6-well plates at a density of 5 × 10^4^ cells per well. After 24 hours of culture, trypsin or amylase activity assays were performed. For trypsin activity, trypsinogen was activated with 1 µL human enteropeptidase (1.4 µg/ml stock, R&D Systems, Minneapolis, MN) for 1 hour at 37°C. Trypsin activity in cultured media was determined using a trypsin activity assay kit (BioVision, Milpitas, CA). A 50 µL aliquot of each sample was mixed with 150 µL 200 µM N-CBZ-Gly-Pro-Arg-p-nitroanilide substrate, and the release of yellow p-nitroaniline was measured at 405 nm using a SpectraMax plus 384 microplate reader (Molecular Devices, Sunnyvale, CA) over a period of 1 minute.

Amylase activity in culture media was measured using an amylase activity colorimetric assay kit (Biovision) according to the manufacturer’s instructions. For serum amylase activity, blood samples from mice were centrifuged at 1,500*g* for 10 mins at 4℃ to separate plasma. Amylase activity in serum was measured using an amylase activity assay kit (E-BC-K006-M, Elabscience) as per the manufacturer's instructions.

*Histologic analysis, immunohistochemistry, immunofluorescence, and TUNEL assay*: Pancreatic tissues were fixed in paraformaldehyde at room temperature for 24 hours, embedded in paraffin, and subjected to H&E staining. Histological examination involved scoring 5 random fields (×100 magnification) for edema, inflammatory cell infiltration, and acinar necrosis by two independent, blinded investigators. Scores ranged from 0 to 4, with results presented as means ± SEM.^[8]^ Sirius red and Masson’s trichrome staining was performed to assess fibrosis.

Immunohistochemistry involved staining pancreatic biopsy sections with anti-alpha smooth muscle actin (α-SMA) polyclonal antibody (GB111364, Servicebio; 1:200), anti-F4/80 polyclonal antibody (GB113373, Servicebio; 1:200), anti-amylase monoclonal antibody (3796, Cell Signaling Technology; 1:1600), and anti-CD45 polyclonal antibody (GB113886, Servicebio; 1:200). Immunofluorescence was used to detect the ductal cell marker cytokeratin 19 (CK19) using monoclonal antibody (ab52625, Abcam; 1:200). Apoptosis levels were assessed using terminal transferase-mediated dUTP nick end-labeling (TUNEL) staining with a TUNEL staining kit (A112, Vazyme).

*Intrapancreatic trypsin activity*: The procedure followed a slightly modified version of a previously described method^[9]^. Briefly, mouse pancreata were homogenized in 1000 μL of trypsin assay buffer and centrifuged to obtain clear extracts. The extracts were then reacted with 1 μL Z-Gly-Pro-Arg-AMC hydrochloride (GPR-AMC) fluorescent substrate (HY-P4217, MedChemExpress) (2 mM) and monitored using a fluorescence plate reader with excitation at 380 nm and emission at 460 nm.

*Transmission electron microscopy (TEM)*: Pancreatic tissue samples (1 mm^3^ pieces) or isolated pancreatic acinar cells were initially fixed in fresh fixative (G1102, Servicebio) for 24 hours at 4℃. Subsequently, they were fixed in 1% OsO_4_ in 0.1 M sodium phosphate buffer for 2 hours, rinsed in the same buffer, and then washed in distilled water for 20 minutes. The specimens underwent dehydration through a graded series of alcohol (30%-100%) and were washed twice with 100% acetone for 15 minutes each. Infiltration was performed with a 1:1 mixture of acetone and EMBed 812 for 30 minutes, followed by a 3:1 acetone-EMBed 812 mixture overnight, and then with fresh EMBed 812 for 6 hours. The embedded molds were cured in a 60℃ oven for 48 hours. Ultrathin sections (50 nm) were prepared and mounted on 150 mesh copper grids. Sections were stained with 2.6% lead citrate and examined using a TEM (HT7800, Hitachi).

*Reverse transcription-PCR and quantitative PCR analyses*: RNA was extracted from both wild-type and mutant HEK293T cells, as well as from the pancreata of wild-type and *Sec16a* knockout mice, using the RNeasy Mini Kit (Qiagen, Germantown, MD). 2 µg RNA were reverse-transcribed with the High-Capacity cDNA Reverse Transcription Kit (Applied Biosystems). For semiquantitative analysis of X-box-binding protein 1 (*XBP1*) mRNA splicing, PCR was performed with primers amplifying both spliced and unspliced forms, yielding 415-bp and 441-bp amplicons, respectively. The PCR products were analyzed by agarose gel electrophoresis.

Quantitative real-time PCR for gene expression analysis was performed using a 7900 Real-Time PCR System (Applied Biosystems). We analyzed genes encoding fibrogenic factors (*Col1a1*, *Fn1*, *Tgf-β*) and a proinflammatory factor (*Il6*), with primers detailed in Table S4. The comparative CT method was employed for quantification: target gene expression was first normalized to the actin control (∆CT), and then to wild-type cell levels (∆∆CT). Results were expressed as fold changes, calculated using the formula 2−∆∆CT.

**Table S1.** Rare *SEC16A* nonsynonymous variants in the French CP cohort.

| Location | Nucleotide  change | Amino acid change | Patients | Allele frequency in gnomAD  (all populations) | CADD score |
| --- | --- | --- | --- | --- | --- |
| **Idiopathic CP (n = 192)** | | | | | |
| **Exon 3** | **c.140C>T** | **p.P47L** | **1 (0.52%)** | 0.01% | **23.5** |
| Exon 3 | c.1081C>T | p.P361S | 2 (1.04%) | 0.08% | 8.2 |
| Exon 3 | c.2237C>T | p.A746V | 1 (0.52%) | 0.01% | 5.1 |
| Exon 3 | c.2243A>C | p.Y748S | 1 (0.52%) | 0.08% | 15.3 |
| Exon 3 | c.2252C>G | p.A751G | 1 (0.52%) | 0.20% | 4.4 |
| Exon 3 | c.2623C>A | p.L875I | 1 (0.52%) | 0.00% | 10.7 |
| Exon 3 | c.2668A>G | p.T890A | 1 (0.52%) | 0.00% | 0.0 |
| **Exon 3** | **c.2977G>A** | **p.A993T** | **2 (1.04%)** | 0.29% | **22.5** |
| Exon 3 | c.2998A>G | p.R1000G | 1 (0.52%) | 0.00% | 13.6 |
| **Exon 5** | **c.3715C>G** | **p.P1239A** | **1 (0.52%)** | 0.00% | **24.2** |
| **Exon 7** | **c.4034G>A^a)^** | **p.R1345Q^a)^** | **1 (0.52%)** | 0.01% | **26.2** |
| **Exon 7** | **c.4063C>T** | **p.R1355W** | **1 (0.52%)** | 0.07% | **26.8** |
| Exon 7 | c.4125C>A | p.H1375Q | 5 (2.60%) | 0.32% | 0.0 |
| **Exon 10** | **c.4493C>T** | **p.P1498L** | **1 (0.52%)** | 0.00% | **25.2** |
| Exon 22 | c.6086C>T | p.P2029L | 1 (0.52%) | 0.05% | 13.2 |
| **Variant carriers in total** | | | 21 (10.94%) |  |  |
| **Familial/hereditary CP (n = 191)** | | | | | |
| Exon 3 | c.962C>T | p.P321L | 1 (0.52%) | 0.87% | 15.2 |
| Exon 3 | c.1019G>A | p.G340E | 1 (0.52%) | 0.12% | 13.2 |
| Exon 3 | c.1081C>T | p.P361S | 1 (0.52%) | 0.08% | 8.2 |
| **Exon 3** | **c.2008T>G** | **p.C670G** | **1 (0.52%)** | 0.03% | **22.5** |
| Exon 3 | c.2243A>C | p.Y748S | 1 (0.52%) | 0.08% | 15.3 |
| Exon 3 | c.2252C>G | p.A751G | 2 (1.05%) | 0.20% | 4.4 |
| **Exon 3** | **c.2977G>A** | **p.A993T** | **2 (1.05%)** | 0.29% | **22.5** |
| **Exon 3** | **c.3448G>C** | **p.G1150R** | **1 (0.52%)** | 0.00% | **22.5** |
| Exon 3 | c.3455C>T | p.P1152L | 1 (0.52%) | 0.00% | 11.3 |
| Exon 4 | c.3605A>G | p.Y1202C | 1 (0.52%) | 0.20% | 17.2 |
| **Exon 7** | **c.4063C>T** | **p.R1355W** | **2 (1.05%)** | 0.07% | **26.8** |
| Exon 3 | c.4125C>A | p.H1375Q | 5 (2.62%) | 0.32% | 0.0 |
| **Exon 14** | **c.5066C>T** | **p.A1689V** | **1 (0.52%)** | 0.03% | **25.2** |
| **Exon 19** | **c.5653C>T** | **p.R1885W** | **2 (1.05%)** | 0.15% | **23.5** |
| **Exon 30** | **c.6901C>T** | **p.R2301C** | **1 (0.52%)** | 0.00% | **25.1** |
| **Variant carriers in total** | | | **23 (12.04%)** |  |  |

Variants with a CADD score of ≥20 are highlighted in bold.

^a)^Variant also found in the Chinese CP cohort.

**Table S2.** Rare *SEC16A* nonsynonymous variants in FREX (last accessed on August 2, 2023).

| Nucleotide  change | Amino acid change | Allele frequency in FREX | Total variant alleles (number of homozygotes)/total alleles in FREX | Allele frequency in gnomAD (all populations) | CADD score |
| --- | --- | --- | --- | --- | --- |
| c.763A>G | p.I255V | 0.000872 | 1(0)/1146 | 0.000008071 | 2.9 |
| c.1016G>A | p.R339Q | 0.000871 | 1(0)/1148 | 0.00005445 | 15.2 |
| c.1019G>A | p.G340E | 0.00261 | 3(0)/1148 | 0.001226 | 13.2 |
| c.1037G>A | p.R346H | 0.00174 | 2(0)/1148 | 0.0007132 | 1.4 |
| c.1193C>T | p.A398V | 0.000871 | 1(0)/1148 | 0.00008954 | 11.4 |
| c.1357G>A | p.G453S | 0.000871 | 1(0)/1148 | 0.00004026 | 0.1 |
| c.1520G>T | p.G507V | 0.00174 | 2(0)/1148 | 0.00005723 | 17.9 |
| c.2243A>C | p.Y748S | 0.000871 | 1(0)/1148 | 0.0007587 | 15.3 |
| c.2252C>G | p.A751G | 0.00174 | 2(0)/1148 | 0.002043 | 4.4 |
| **c.2506A>G** | **p.S836G** | **0.000871** | **1(0)/1148** | **0.00003616** | **24.3** |
| **c.2977G>A** | **p.A993T** | **0.00522** | **6(0)/1148** | **0.002902** | **22.5** |
| **c.4894G>T** | **p.D1632Y** | **0.000871** | **1(0)/1148** | **0** | **34** |
| **c.5441C>T** | **p.A1814V** | **0.000871** | **1(0)/1148** | **0.00004734** | **24.8** |
| c.6209G>A | p.R2070H | 0.000871 | 1(0)/1148 | 0.0001113 | 0.6 |
| c.7016C>A | p.T2339N | 0.000871 | 1(0)/1148 | 0.00001614 | 13.1 |
| **Variant alleles in total** | | 0.021777 | 25(0)/1148 |  |  |

Only high-quality missense, nonsense, small indel or canonical splice site variants were included for analysis. Rare variants were defined as having an allele frequency of <1% in either FREX or gnomAD. Variants with a CADD scores of >20 are highlighted in bold. The French Exome (FREX) Project comprised whole-exome sequencing data from 574 French subjects.

**Table S3.** Rare *SEC16A* nonsynonymous variants in three publicly available biobanks.

| Nucleotide change  (NM_014866.2) | Amino acid change | Allele frequency in patients | | Allele frequency in controls | | CADD score |
| --- | --- | --- | --- | --- | --- | --- |
|  |  | + | % | + | % |  |
| **Finnish Biobank (3,320 patients [6,640 alleles], 330,903 controls [661,806 alleles])** | | | | | | |
| c.506C>T | p.T169M | 2 | 0.03 | 427 | 0.06 | 0.9 |
| c.898A>G | p.S300G | 13 | 0.20 | 1,002 | 0.15 | 13.6 |
| c.1105_1113del | p.S369_A371del | 16 | 0.24 | 1,769 | 0.27 | 8.8 |
| **c.1883G>A** | **p.R628H** | **32** | **0.48** | **3,022** | **0.46** | **22.5** |
| c.1921C>T | p.R641C | 2 | 0.03 | 284 | 0.04 | 8.3 |
| c.2252C>G | p.A751G | 3 | 0.05 | 132 | 0.02 | 4.4 |
| **c.2977G>A** | **p.A993T** | **5** | **0.08** | **503** | **0.08** | **22.5** |
| c.2989A>G | p.T997A | 49 | 0.74 | 4,073 | 0.62 | 0.004 |
| c.3185T>C | p.V1062A | 49 | 0.74 | 4,073 | 0.62 | 0.064 |
| **c.4063C>T** | **p.R1355W** | **40** | **0.60** | **2,805** | **0.42** | **26.8** |
| c.4288C>T | p.P1430S | 1 | 0.02 | 144 | 0.02 | 11.1 |
| **c.4718C>T** | **p.S1573L** | **2** | **0.03** | **156** | **0.02** | **27.8** |
| **c.5050G>A** | **p.G1684R** | **5** | **0.08** | **419** | **0.06** | **28** |
| **c.5266A>G** | **p.K1756E** | **2** | **0.03** | **183** | **0.03** | **26.2** |
| **c.5653C>T** | **p.R1885W** | **5** | **0.08** | **594** | **0.09** | **23.5** |
| c.5828C>T | p.S1943L | 3 | 0.05 | 257 | 0.04 | 3.0 |
| c.6086C>T | p.P2029L | 4 | 0.06 | 252 | 0.04 | 13.2 |
| c.6533C>T | p.P2178L | 1 | 0.02 | 24 | 3.63×10^-3^ | 17.4 |
| **c.6874C>G** | **p.R2292G** | **2** | **0.03** | **207** | **0.03** | **31** |
| **c.6988C>T** | **p.P2330S** | **1** | **0.02** | **90** | **0.01** | **27.2** |
| **Variant alleles in total** | | **237** | **3.57** | **20,416** | **3.08** |  |
| **UK Biobank (607 patients [1,214 alleles], 417,106 controls [834,212 alleles])** | | | | | | |
| c.506C>T | p.T169M | 19 | 1.57 | 8,051 | 0.97 | 0.9 |
| c.1019G>A | p.G340E | 4 | 0.33 | 2,612 | 0.31 | 13.2 |
| c.1105_1113del | p.S369_A371del | 8 | 0.66 | 6,101 | 0.73 | 8.8 |
| c.2252C>G | p.A751G | 6 | 0.49 | 2,414 | 0.29 | 4.4 |
| **c.2977G>A** | **p.A993T** | **4** | **0.33** | **5,028** | **0.60** | **22.5** |
| **c.5653C>T** | **p.R1885W** | **4** | **0.33** | **2,080** | **0.25** | **23.5** |
| **Variant alleles in total** | | **45** | **3.71** | **26,286** | **3.15** |  |
| **Japan Biobank (457 patients [914 alleles], 177,471 controls [354,942 alleles])** | | | | | | |
| c.752C>G | p.P251R | 2 | 0.22 | 610 | 0.17 | 12.9 |
| c.815C>T | p.P272L | 2 | 0.22 | 1,481 | 0.42 | 14.7 |
| **c.1036C>T** | **p.R346C** | **1** | **0.11** | **264** | **0.07** | **22.6** |
| c.4247C>T | p.P1416L | 5 | 0.55 | 720 | 0.20 | 9.8 |
| c.4270C>T | p.P1424S | 0 | 0.00 | 271 | 0.08 | 18.3 |
| **c.6616G>A** | **p.E2206K** | **5** | **0.55** | **1,677** | **0.47** | **22.1** |
| **c.6628G>A** | **p.A2210T** | **0** | **0.00** | **128** | **0.04** | **21.7** |
| c.6740G>A | p.G2247E | 3 | 0.33 | 1,108 | 0.31 | 18.5 |
| **Variant alleles in total** | | **18** | **1.97** | **6,259** | **1.76** |  |

Variants with a CADD scores of >20 are highlighted in bold.

**Table S4.** Primers for targeted next-generation sequencing of the *SEC16A* gene.

| **Region** | **Forward primer sequences (5' to 3')** | **Reverse primer sequences (5' to 3')** |
| --- | --- | --- |
| Exon 3 | CTGTAAGCAGCATCGACTTGTG | GCAAGACAGGTGGACTGCTTTT |
| Exon 3 | TAGTAGACAGGCGCTCCAAAGTA | CTCTGCACTCCTGTTCATCTCAG |
| Exon 3 | TTCTGGTGCATTGACACCTTCA | CACCGTCATGTGGGTTTTGC |
| Exon 3 | CCCTCATGGGAACATGCCTG | GGCCCTGATGTAGGATGGAC |
| Exon 3 | GCCACCAGCGTTCCTCATT | GCTTGCCCAGTGATTCACAATT |
| Exon 3 | AATAACTCTGATCCTGAAAGTACATTCAGG | GAAGCTCCTGAGTCTGCTTCTAG |
| Exon 3 | GGAGATAGCCCAGAAAACCGTAC | GCTGGAGCAGAAATCGTCAAAG |
| Exon 3 | CTCTCATCTGAAAAAGCAGGCTTATCT | GCTCACTTGGCAGAACTTCTTG |
| Exon 3 | CGCAGTATGAGAATGTTGAGAACTTAGAAT | GCTTCTGCTGCTATAGCTGGAT |
| Exon 3 | CCGAGTGACCAGTTCAGATATGG | TCACCTCCTACGGGAGAAGAATC |
| Exon 3 | CGAGGATGAAGCTTCAGGTAGTTTTT | GTCTCCCTTACTTCACCAACCA |
| Exon 3 | GGGTAAAACCATTTGAGGCAGA | TCGGGTGCTGGATACACAGTAT |
| Exon 3 | GGAAGCTGTGCACATGCTTC | CCTCTTCTGGAGGCTGAACA |
| Exon 3 | CGCTTTATGTGTGTGCAAAACCT | ACTGGAGGATTCTGCAGAGACT |
| Exon 3 | GAGTTCTGGTTATGCAAGTTTATTATCCTC | AGCCTCTCTCCAGGACTGATTC |
| Exon 3 | CCATTAACTTTTCTGTGTCCTTATCGAAC | GATGGTGGTTGAACCAGCAAAT |
| Exon 3 | TGGTGCTTCCGAAATGGTTTCTAA | TTAAGGTAAAGTCTAGGGCTCCGTAA |
| Exon 3 | GGTAATAAGGCAAACCATTCCAGTCAT | CATCTTTCGTGACCTGCTGATAAAAAC |
| Exon 3 | GCCAGTCATCCCAGACAATCTG | GTCGACCAGAACCAGACTTGCT |
| Exon 3 | GAGCTGTCAAATCCAGAAAGTCTG | GGCTGGTAGGCATCGTACAAAG |
| Exon 3 | CTCAGTCCTCTAGCGTGTCTCT | TCAACTGGTCAAAGCACAGACA |
| Exon 4 | CGAGCTTGCTCTTGATTGGTGT | CCATAGCAAGCAACACAGACATG |
| Exon 5 | GAATGATTTGTATTGGCTGATGTCACAC | GGCTGCATGTCATCAGTGTCTT |
| Exon 6 | CGCTTCTTTAGTGATATCTGAGGCAT | CATGAGGGCAGAGAAGCACTAG |
| Exon 7 | CTTCTCTAACGTTGCACGTCAGA | GTGAAGCGAGGATCGTACCT |
| Exon 7 | CCGAGAAACGTGACAACAACTG | AAAACTTTAGGTACATTGCAAACAGGATG |
| Exon 8 | GGAATTGATTTTGCCCGTTTGCTT | GGTTAGCCGGTATCCCTCTGT |
| Exon 9 | GGCTCAGAAGCATCAGTGACAA | ATGACAAACTGCAAAGCAGAGAGT |
| Exon 10 | GCCATCCTCTCCTCTCACG | ATTAATGACATCCACCTTATGGGTGTC |
| Exon 11 | CCTTGTTGAGGCATTTCTTTGTGAG | TGAGCATTCCCAAGACGAAATGA |
| Exon 12 | CCCTTAGGAAGCACACTGTAGTG | CGTAACAGAAGCTCCGCAATGT |
| Exon 12 | TGTTCCTGTTGACCCAAACAGAC | CCTTCTTACGGCCATACAGCAA |
| Exon 12 | CTCGAGAGAGAGACCGAGAGGTT | CAACACTGGAAACAGTTAGAAGGAAAC |
| Exon 13 | CTTTGGGTTGTTTGTGTATCTGCT | CAGGCCATTCTTCATTGCAGAC |
| Exon 13 | GTCCCTGAAATGCCTTTTGCAG | GTGGGAGCACAGCACAGTCAAG |
| Exon 14 | CATGTTAGTCTCTTAACTTCTGACCTCA | GGACATGAGCTGGTAGACTGTCT |
| Exon 14 | TTGCTAACAGCCTCCCAATCAA | CATTTCTCGTCTCCACAGCACTA |
| Exon 15 | CCCAGCTGTGTGGAATTGAAATG | TTCTGCCAACGCCACAGACAAC |
| Exon 16 | CGTTGGCAGAAATCACCAGAAC | TACAGGACTACACTGACTTGGTTCT |
| Exon 16 | ACAAAGCTTGTCTTAATCGGATCCA | GCTTCGTTGGTTGCGAACTTTA |
| Exon 17 | TTCTGGATGATTTGCTCCAAGGTT | CAATTCAAACAATCTAAGTAGCCAAAAGCT |
| Exon 18 | CCAGAAGAGTTCACAGCTCACA | GCGGCAGGAGTAGATGAACTTA |
| Exon 18 | CAGCAAGGCTTCTGTCGTTTC | TACCACAATGCGCGTCTGAA |
| Exon 19 | GCATTTTGAAGACGGCTCACTT | CAGATACATACTTTCAGTGTGGAAATGACA |
| Exon 20 | CTGAAGTAGGGACACAGCAAGC | GCTCCTGGCTGACTGAGTC |
| Exon 20 | GAGTTCCGAGATGGAGCAGTT | GCCTCCAACAGTCCACATCTG |
| Exon 21 | CAGTAACTCAAGCTACCCTCAGTAAC | CCTGGAGCAAGTGTCTCCTTTC |
| Exon 21 | GAGTGCCGATGTTCCCAGT | TCAAGCTCAAATAACTTCAATCTCTTCCA |
| Exon 22 | AGCCTTGGTTTCTGCAGATCAT | TGCCCACTCGATGAACTTTATGG |
| Exon 23 | AGGAGAGCCTGTTCTGCATTTC | GTTCCTTCGTTTCTTTCTTGGCT |
| Exon 23 | CTCACCCGCTCCCGAAACAAAG | CTGACCTGGGTGGGCTATTATC |
| Exon 24 | CTCACAGAAGTGGGTCATGTAGAAT | TTCAAAACTTCAGGAAACACCTACTCA |
| Exon 25 | GTGTTTCCTGAAGTTTTGAAGTGTCATC | GCAACAGCCACCCAAATATCAC |
| Exon 26 | CCTGTGATATTTGGGTGGCTGTT | TCTCTCTGGGACAGTTAATCGTTCA |
| Exon 27 | CGCGTTCACCTCAATTTGCTTC | TCATTCTTTTAACGGGAGATTTAGGAGAG |
| Exon 28 | GATTTGCCTGTTTCTGCATGAGT | AGCTGTGGTTCTTCTGCATCTG |
| Exon 28 | TCTCGGAGTGTCTTCAGTGAGA | CGTACACATTGGATACTGACTTGCA |
| Exon 29 | TGGCCTGGTGTGAGAGACTTAA | ACCTTCGAGGTGCTCTTGTTTC |
| Exon 30 | GACGTGCATGTTGACTTTCGTC | CGCCAGCTCTCATGTTAGTGAG |
| Exon 31 | CCAGAAGCGGTGTCTGTATCTG | GAAAGAAAAAGAGGATCTAGGGCAGAA |
| Exon 32 | CCGTGTGGTTCATTATGTGGAC | TTCACAGCAGGGCAAGCCTAGT |
| Exon 32 | CTAGGGAGGATTGGCCAGAGGAA | CAATTCTGAGTCAAAGATTCAGTCTTTCC |

**Table S5.** Primers for Sanger sequencing of the *SEC16A* gene.

| **Region** | **Forward primer sequences (5' to 3')** | **Reverse primer sequences (5' to 3')** |
| --- | --- | --- |
| Exon 3 | GGGTGTAGGGGAGAGAGAGT | ATGCACCAGAAAACGGACTG |
|  | CGGTTTGCTTGTTCCTCACA | CACTCCTGGATTCTGCCTGA |
|  | CCACCTGGCCAATAACTCTG | GGCTCACTTGGCAGAACTTC |
|  | CAATGAGGCTGCTGGTGATG | CCCTTACTTCACCAACCACG |
|  | GTGAGCCAGAATTACCGTGG | GCATAACCAGAACTCGCCTG |
|  | TGTGTGTGCAAAACCTCAGC | TAGCAAATCCGGGAAGAGCA |
|  | TTCCAACCAGCTCTGTCCTT | TGGTGACTGTGCTGAGTTCT |
|  | CCCAAAGCCATGTTTTCGGA | GGAGAGTAGGTTCTGGAGCC |
| Exon 4 | GTGCTTTGGAGGTCTGTGTG | AGCAAGCAACACAGACATGG |
| Exon 5 | TCCACAGTCTCATCCTTGCT | CAGGGCTGCATGTCATCAG |
| Exon 6 | TAAAGTCCTGGGGTGGTGTC | GTCTGCTCCCTCCTTCCTTT |
| Exons 7 and 8 | CTCCCATTCCTCTGCCTCTG | AATTCCAATCCCAGCCCTGA |
| Exon 9 | TGAGTTGGAAGTCTGTCGTGA | AACCACGTTGAACACCCAAG |
| Exons 10 and 11 | CCATGCCATCCTCTCCTCTC | ACAGTGTGCTTCCTAAGGGG |
| Exon 12 | TTCGGGTAGTTGTGGGTCTG | GGAAACAGCAAGACCCGAAG |
| Exon 13 | TGGCCTGTCTTGACTCATGT | CACAACACGACCCAAGGAAG |
| Exons 14 and 15 | CAGCATGGGGTCTTTCTGTG | CATCTCCACCCCTGACCTAC |
| Exons 16 and 17 | GCGTCCTTGACTTCTGCAA | TGCAAACGGAAAGCCTCAAT |
| Exon 18 | GGTGGGAGACGATGATGAGT | AGGCTAAGACGCAAAACACG |
| Exons 19 and 20 | TTTGAAGACGGCTCACTTGC | TCAGGGGAGGAAGCATGAAG |
| Exon 21 | GAGTGTCAGCTCCTTCCAGT | TCTCTTCCACCAATCCCCAC |
| Exon 22 | GGTGTGCCTGTTGATCTGTG | TCTTCCCCATCTTTCCCTGC |
| Exon 23 | CCGGCCTCTCAGTGATGTAT | ACTGTAGACACTCGGCTGTT |
| Exons 24 to 26 | CTCTGTGGATCTGGCGACTC | ACAGCTACACATTGGCCTCT |
| Exons 27 and 28 | AGAGGCCAATGTGTAGCTGT | TACCTTTCCCCTTTCCCACC |
| Exon 29 | TAACCCAGGCCAGAACAGTT | GCCTCTTCCTACCCAGTACC |
| Exon 30 | ACAGTGACGTGCATGTTGAC | TCACACCATTGCATTCCAGC |
| Exon 31 | GAAGCGGTGTCTGTATCTGC | CGTGTTAGGGTCTCGTAGGG |
| Exon 32 | GCACTGACTTGTCGGTTTCC | CCAGCTCTGAGTCACTGCT |

**Table S6.** Single guide RNA (sgRNA) sequences for the *CRISPR/Cas9* system.

| **Region** | **Nucleotide**  **change†** | **Amino acid change** | **Reverse primer sequences (5' to 3')** |
| --- | --- | --- | --- |
| Exon 10 | c.4484G>A | p.R1495Q | CATACTTGGCCAGGGGTCCC |
| Exon 19 | c.5839G>T | p.V1947L | AGCACTCGAGCCCGAGCGTG |
| Exon 3 | c.3115C>T | p.R1039C | TCGTGACCTGCTGATAAAAA |

**Table S7.** Primers for quantitative reverse transcription PCR analysis of *Xbp1*, *Col1a1*, *Fn1*, *Il6* and *Tgf-β* mRNAs.

| **Target** | **Forward primer sequences (5' to 3')** | **Reverse primer sequences (5' to 3')** |
| --- | --- | --- |
| *Xbp1* | CCTTGTAG TTGAGAACCAGG | GGGCTTGGTATATATGTGG |
| *Col1a1* | ATGACGTGCAATGCAATGAAGA | CCATAGGACATCTGGGAAGCAA |
| *Fn1* | ACCCGTGAAGAATGAAGAGGA' | CGTGATGGCGGATGATGTAG |
| *Il6* | GTCCTTCCTACCCCAATTTCCA | CGCACTAGGTTTGCCGAGTA |
| *Tgf-β* | GAGGCGGTGCTCGCTTTGTA | TTCCCGAATGTCTGACGTATTGA |


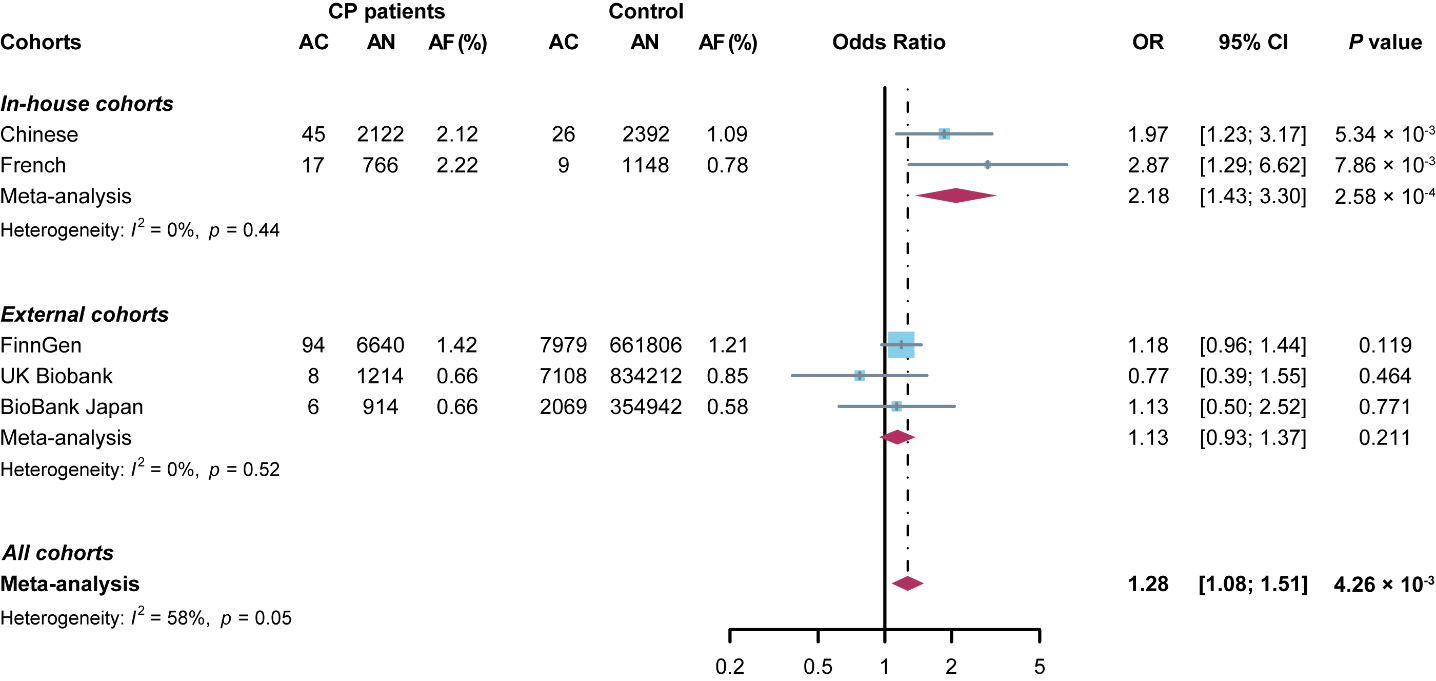


**Figure S1.** Meta-analysis of rare *SEC16A* variants with a CADD score of ≥20 in Chinese CP discovery cohort and replication cohorts. CP, chronic pancreatitis; AC, allele count; AN, allele number; AF, allele frequency; OR, odds ratio; CI, confidence interval.


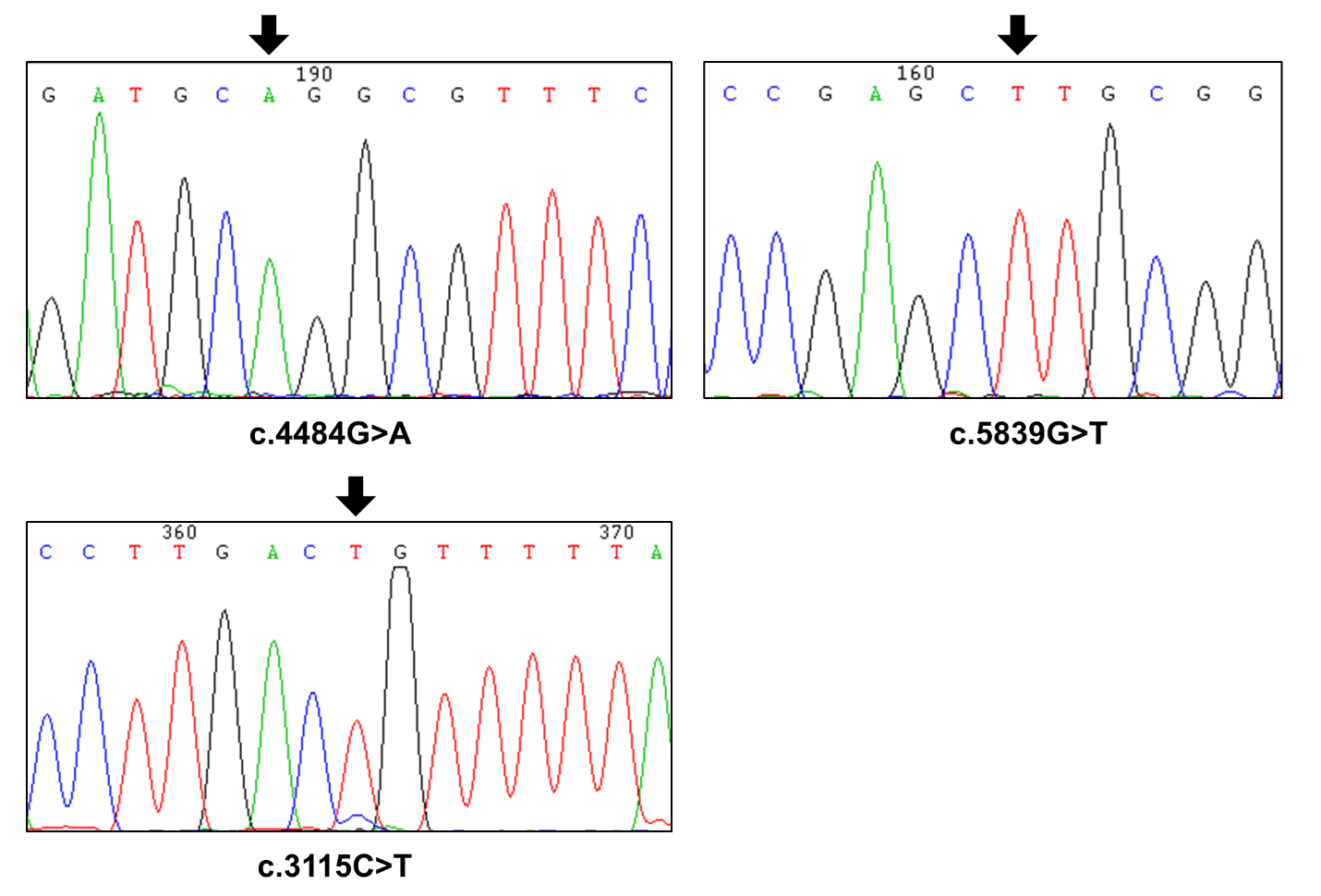


**Figure S2.** Sanger sequencing validation of three Cas9-edited HEK293T cell lines with indicated *SEC16A* mutations.


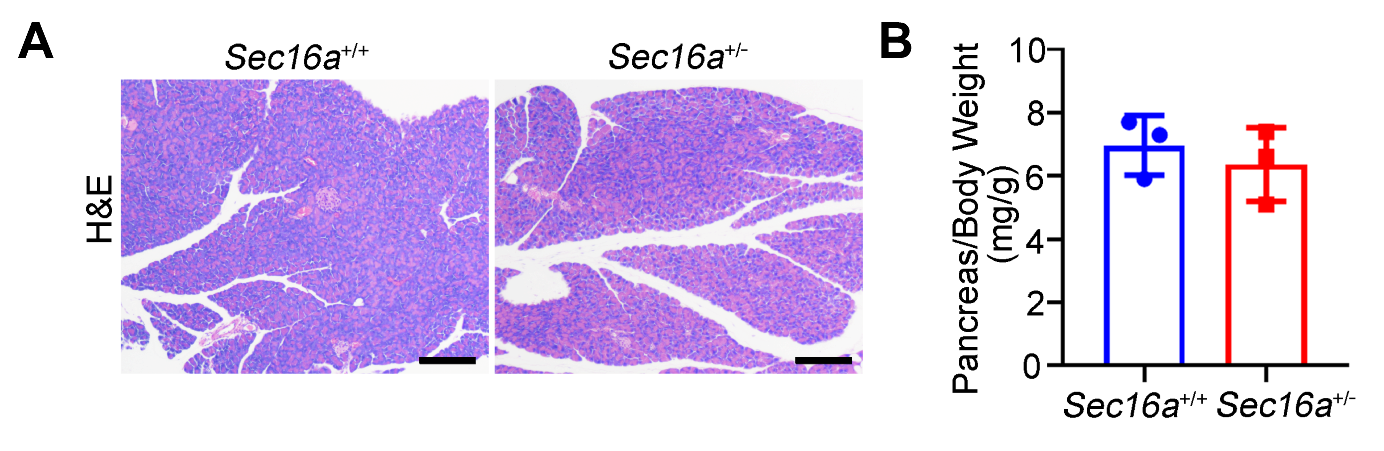


**Figure S3.** Absence of pathological changes in *Sec16a^+/-^* mice 8 weeks after birth. A) Representative H&E-stained pancreas sections of *Sec16a^+/+^* and *Sec16a^+/-^* mice at 8 weeks of age. Scale bar: 200μm. B) Pancreas/body weight ratio of *Sec16a^+/+^* and *Sec16a^+/-^* mice at 8 weeks of age. Data are presented as mean ± SD, analyzed using a two-tailed unpaired Student’s *t* test.


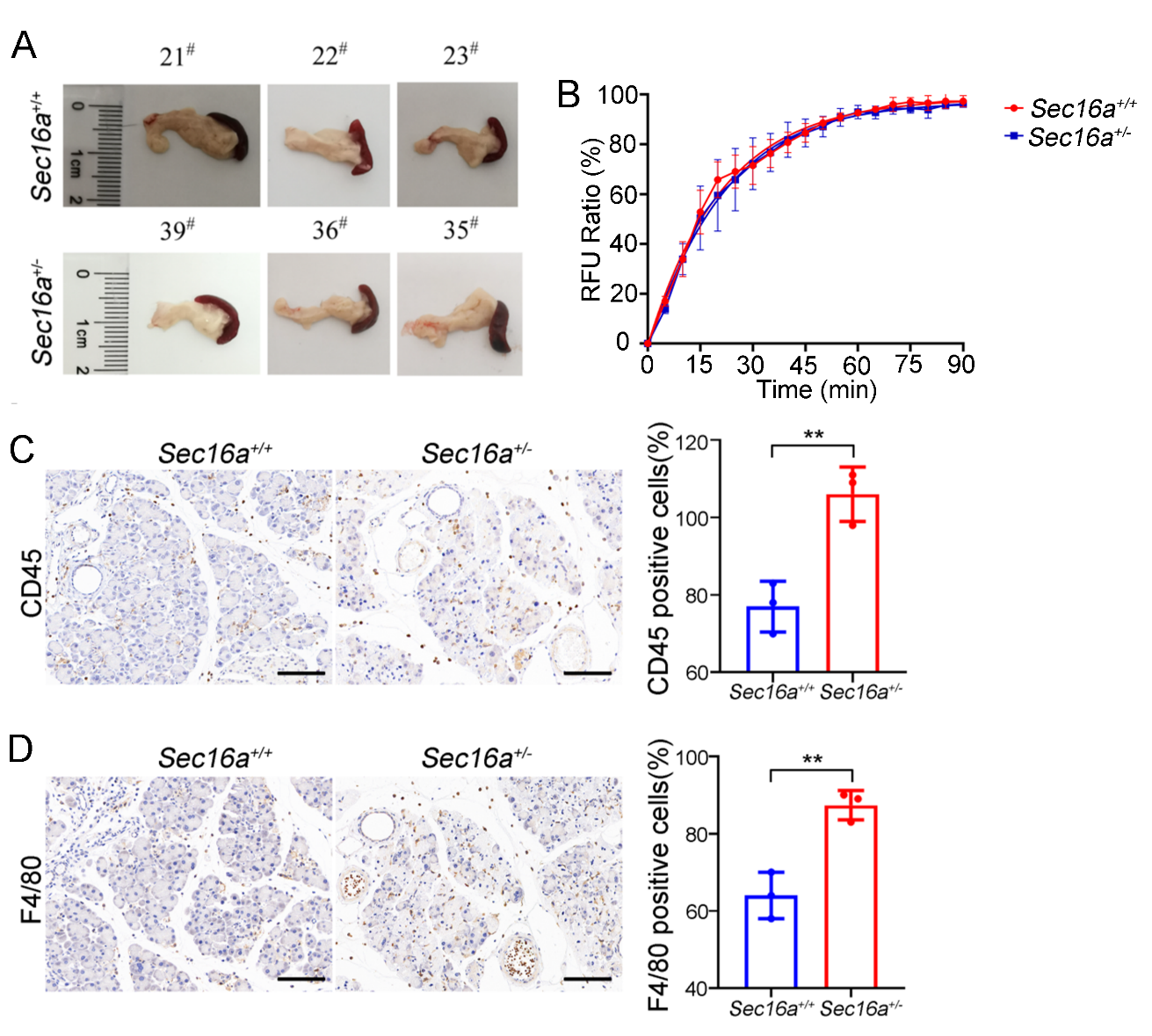


**Figure S4.** Cerulein-induced AP in *Sec16a^+/+^* and *Sec16a^+/-^* mice. A) Representative pancreas images showing AP in *Sec16a^+/+^* and *Sec16a^+/-^* mice. B) No significant differences in intrapancreatic trypsin activity were observed between *Sec16a^+/-^* and *Sec16a^+/+^* mice in cerulein-stimulated AP models. Relative fluorescent units (RFU) ratios in each group were measured relative to the maximal RFU. Data from three experiments were globally fitted. C) Representative immunohistochemical staining and quantification of CD45-positive inflammatory leukocytes (brown signal). Scale bar: 100 μm. D) Representative immunohistochemical staining and quantification of F4/80-positive inflammatory macrophages (brown signal). Scale bar: 100 μm. Data are expressed as mean ± SD. Statistical significance was assessed using a two-tailed unpaired Student’s *t*-test, with ^**^ indicating a *P* value of < 0.01.


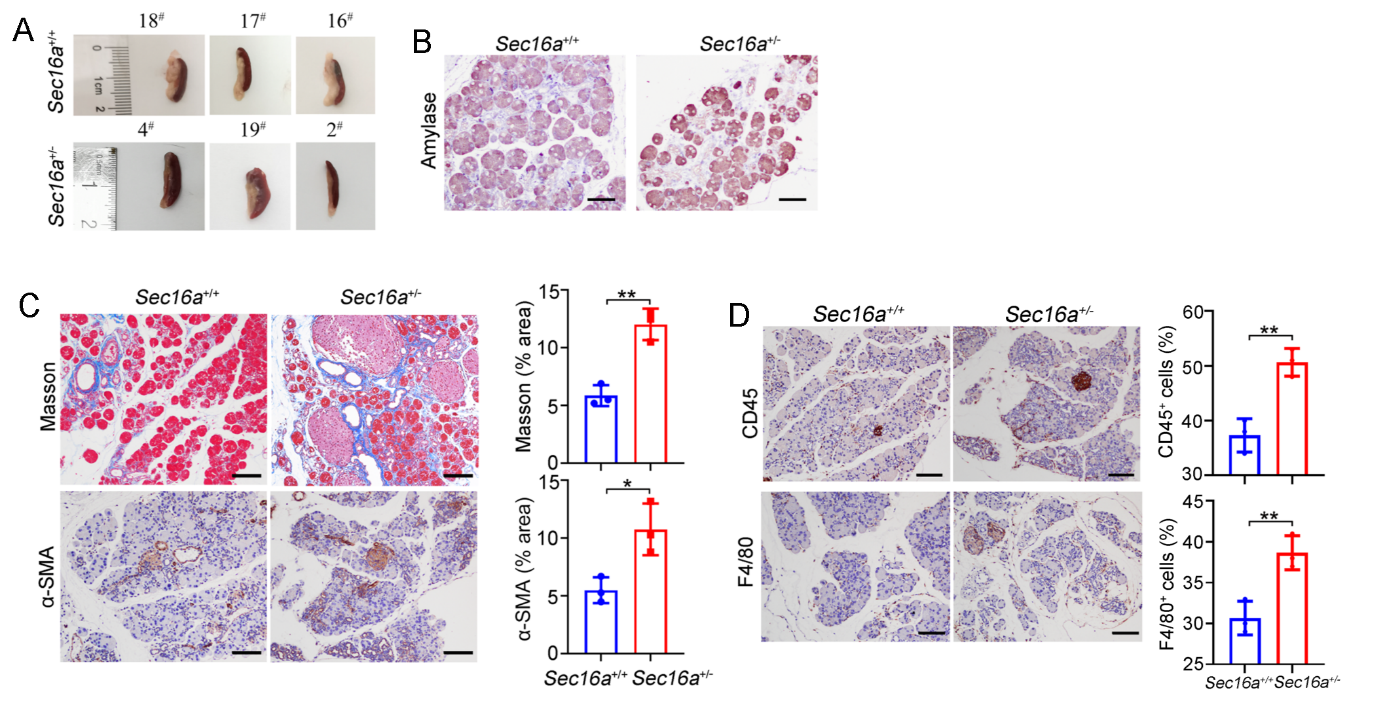


**Figure S5.** Cerulein-induced CP in *Sec16a^+/+^* and *Sec16a^+/-^* mice. A) Representative pancreas images demonstrating CP in *Sec16a^+/+^* and *Sec16a^+/-^* mice. B) Immunohistochemical staining for amylase in pancreatic tissues from *Sec16a^+/+^* and *Sec16a^+/-^* mice subjected to cerulein-induced CP. C) Masson’s trichrome staining and immunohistochemical staining for α-SMA (left-hand panel), and the percentage positive area for fibrosis and α-SMA (right-hand graph). D) Representative immunohistochemical staining and quantification of CD45-positive leukocytes and F4/80-positive macrophages (brown signal). Scale bar: 100 μm. Data are presented as mean ± SD. ^*^, *P* < 0.05; ^**^, *P* < 0.01. Two-tailed unpaired Student’s *t* test for column analysis; two-way ANOVA with Tukey’s test for grouped analysis.

**References**

[1] M. Li, Z. Zhang, X. Li, J. Ye, X. Wu, Z. Tan, C. Liu, B. Shen, X. A. Wang, W. Wu, D. Zhou, D. Zhang, T. Wang, B. Liu, K. Qu, Q. Ding, H. Weng, Q. Ding, J. Mu, Y. Shu, R. Bao, Y. Cao, P. Chen, T. Liu, L. Jiang, Y. Hu, P. Dong, J. Gu, W. Lu, W. Shi, J. Lu, W. Gong, Z. Tang, Y. Zhang, X. Wang, Y. E. Chin, X. Weng, H. Zhang, W. Tang, Y. Zheng, L. He, H. Wang, Y. Liu, Y. Liu, *Nat Genet*. **2014**, 46(8): 872.

[2] T. Forshew, M. Murtaza, C. Parkinson, D. Gale, D. W. Tsui, F. Kaper, S. J. Dawson, A. M. Piskorz, M. Jimenez-Linan, D. Bentley, J. Hadfield, A. P. May, C. Caldas, J. D. Brenton, N. Rosenfeld, *Sci Transl Med*. **2012**, 4(136): 136ra68.

[3] H. Wu, D. Z. Zhou, D. Berki, A. Geisz, W. B. Zou, X. T. Sun, L. H. Hu, Z. H. Zhao, A. J. Zhao, L. He, D. N. Cooper, C. Ferec, J. M. Chen, Z. S. Li, M. Sahin-Toth, Z. Liao, *Hum Mutat*. **2017**, 38(8): 959.

[4] S. Hamada, E. Masson, J. M. Chen, R. Sakaguchi, V. Rebours, L. Buscail, R. Matsumoto, Y. Tanaka, K. Kikuta, F. Kataoka, A. Sasaki, M. Le Rhun, H. Audin, A. Lachaux, B. Caumont, D. Lorenzo, K. Billiemaz, R. Besnard, S. Koch, T. Lamireau, X. De Koninck, E. Génin, D. N. Cooper, Y. Mori, A. Masamune, C. Férec, *Hum Mutat*. **2022**, 43(2): 228.

[5] L. Wen, T. A. Javed, D. Yimlamai, A. Mukherjee, X. Xiao, S. Z. Husain, *Gastroenterology*. **2018**, 155(4): 1250.

[6] C. Niederau, L. D. Ferrell, J. H. Grendell, *Gastroenterology*. **1985**, 88(5 Pt 1).

[7] M. M. Lerch, F. S. Gorelick, *Gastroenterology*. **2013**, 144(6): 1180.

[8] J. Schmidt, D. W. Rattner, K. Lewandrowski, C. C. Compton, U. Mandavilli, W. T. Knoefel, A. L. Warshaw, *Ann Surg*. **1992**, 215(1): 44.

[9] Y. C. Wang, X. T. Mao, C. Sun, Y. H. Wang, Y. Z. Zheng, S. H. Xiong, M. Y. Liu, S. H. Mao, Q. W. Wang, G. X. Ma, D. Wu, Z. S. Li, J. M. Chen, W. B. Zou, Z. Liao, *Gut*. **2024**, 73(7):1142.
